# The *in vitro* host cell immune response to bovine-adapted *Staphylococcus aureus* varies according to bacterial lineage

**DOI:** 10.1038/s41598-019-42424-2

**Published:** 2019-04-16

**Authors:** Mark P. Murphy, Dagmara A. Niedziela, Finola C. Leonard, Orla M. Keane

**Affiliations:** 1 Animal & Bioscience Department, Teagasc, Grange, Dunsany, Co. Meath Ireland; 2 School of Veterinary Medicine, University College Dublin, Belfield, Ireland; 3 Present Address: Department of Medicine, Royal College of Surgeons in Ireland, Dublin, Ireland

## Abstract

Mastitis is the most economically important disease affecting dairy cattle worldwide. *Staphylococcus aureus* is a highly prevalent cause of mastitis, causing infections ranging from sub-clinical to gangrenous. However, the interaction between the genotype of the infecting strain of *S*. *aureus* and the host response remains largely uncharacterised. To better understand the variation in presentation and outcomes of *S*. *aureus*-mediated bovine mastitis, we studied the interaction of a panel of mastitis isolates from several prominent bovine-associated lineages with bovine mammary epithelial cells (bMEC) and neutrophils. Significant differences in immune gene expression by infected primary or immortalised bMEC, or their elaboration of neutrophil chemoattractants, were observed and were dependent on the lineage of the infecting strain. Differences were also apparent in the invasiveness of *S*. *aureus* strains and their ability to survive killing by neutrophils. Our results demonstrate that a range of immune responses occur, suggesting the importance of *S*. *aureus* strain in dictating mastitis disease course. *S*. *aureus* lineages may therefore have adopted differing strategies for exploitation of the intramammary niche. Consequently, improved diagnosis of infecting lineage may enable better prognosis for *S*. *aureus* mastitis and reduce morbidity and economic loss.

## Introduction

Mastitis, commonly due to intramammary infection (IMI), occurs in dairy herds globally. Often requiring antibiotic intervention, it is a burden both to the wellbeing of the animal and the economic output of the herd through a reduction in milk yield, withholding of milk from antibiotic-treated cows and culling of animals in severe cases^1^. While the implementation of mastitis control programs at farm level has reduced the incidence of IMI in recent decades, it remains a significant problem in dairy production. A recent estimate of the cost of clinical mastitis occurring during the first 30 days of lactation in US herds suggests a loss per case of $444 with long-term indirect costs accounting for the majority of the losses^2^.

A wide range of microbial species have been reported to cause bovine IMI although a limited number account for the majority of cases. *Staphylococcus aureus* is among the major pathogens and is the most commonly isolated microorganism from cases of sub-clinical and clinical mastitis in Ireland, accounting for 20–30% of such cases^3,4^. A model of the economic cost of *S*. *aureus* IMI indicated that losses may be as high as €570 per cow infected, substantially higher than for other pathogens^5^.

IMI control strategies often include monitoring milk for somatic cell count (SCC), which mainly reflects the number of leukocytes in the udder. In the case of *S*. *aureus* infections in particular, milk-recording often reveals elevated SCC in animals otherwise lacking in clinical signs of IMI as this pathogen can persist in the intramammary environment. By contrast, infections with *Escherichia coli* are more typically acute and clinical in nature, clearing within a few days^6^. The molecular mechanism underlying the species-specific immune response to these important IMI-associated pathogens has been extensively investigated. Infection of bovine mammary epithelial cells (bMEC) with *E*. *coli* initiates Toll-like receptor (TLR) signalling, resulting in increased levels of active NF-κB and induction of a strong cytokine response. In contrast, *S*. *aureus* infection may fail to activate NF-κB transcription factors resulting in a muted cytokine, and hence SCC, response^7,8^.

Isolates of *S*. *aureus* associated with bovine mastitis predominantly belong to a number of genetically distinct bovine-adapted lineages^9^. Each lineage encodes a diverse assemblage of regulators and effectors of virulence which could individually or additively influence the host immune response and presentation of mastitis^10^. While inter-animal variation plays a role in determining *S*. *aureus* mastitis susceptibility, *S*. *aureus* strains and lineages also differ in their ability to form biofilm^11,12^, coagulate plasma, produce toxins^13^, and elicit an immune response from bMEC^14,15^. Failure to elicit a robust local pro-inflammatory response in bMECs that would result in attraction of immune cells to the site of infection could have important consequences for mastitis presentation and diagnosis.

The major lineages associated with IMI in Ireland are CC71, CC97, ST136 and CC151^16^. In this study, we examined *in vitro* the potential for lineage-specific virulence of a panel of strains of *S*. *aureus* in their interactions with the MAC-T immortalised bovine mammary epithelial cell line, as well as primary bMEC and neutrophils. These strains were isolated from cases of clinical mastitis in Ireland^4^ and comprised three isolates from each of the four major lineages. The ability of the strains to induce a pro-inflammatory immune response from bovine mammary epithelial cells resulting in neutrophil chemotaxis, as well as their propensity to invade bMEC, were characterised. We additionally examined survival of the strains during incubation with bovine granulocytes.

## Results

### Expression of pro-inflammatory immune genes differs by lineage of infecting strain

**Figure 1:**
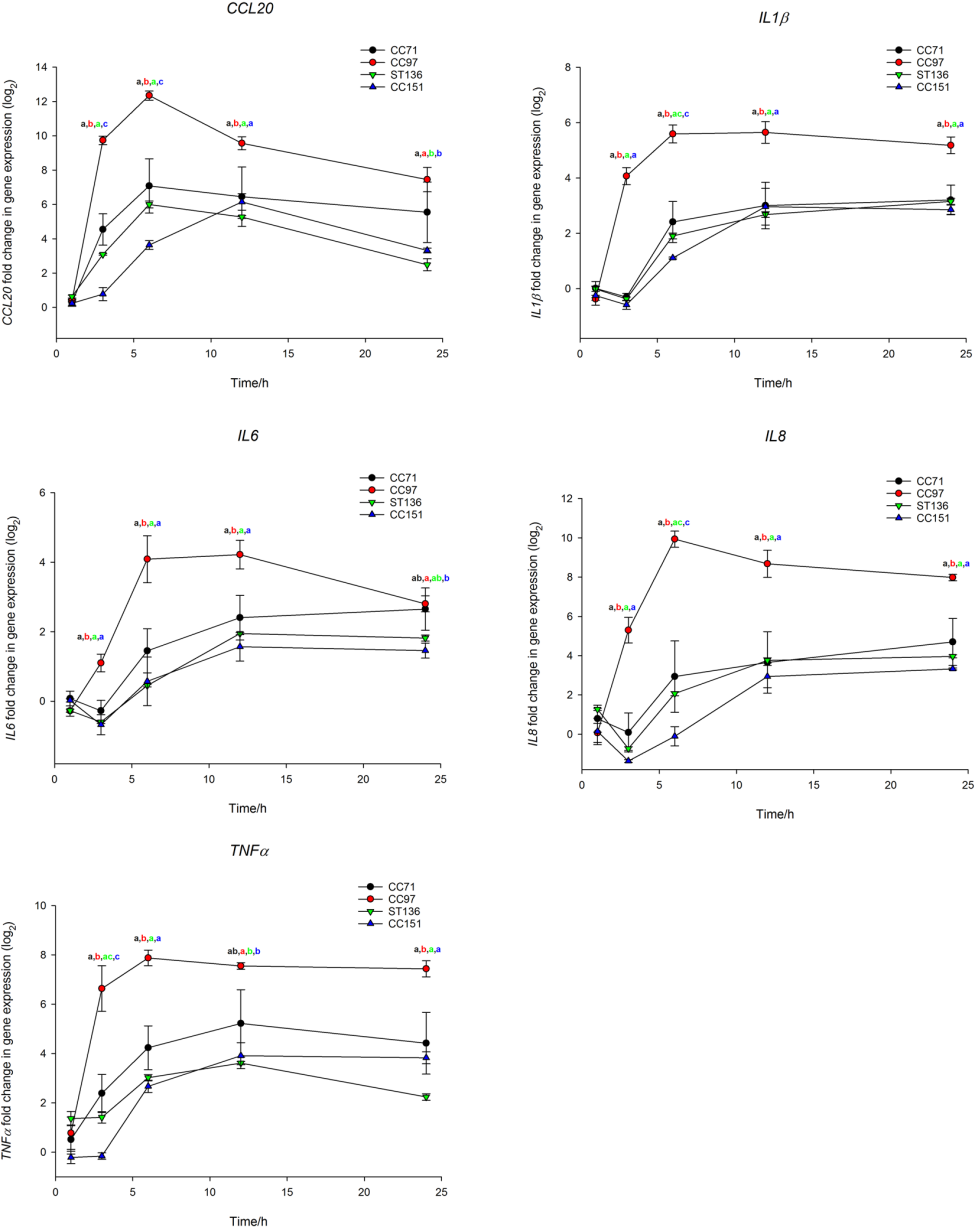
Gene expression in the MAC-T bovine mammary epithelial cell line in response to infection with *S*. *aureus* from bovine-adapted lineages. Expression of (**A**) *CCL20*, (**B**) *IL1β*, (**C**) *IL6*, (**D**) *IL8* and (**E**) *TNFα* over time in MAC-T cells infected with strains from *S*. *aureus* lineages CC71 (black), CC97 (red), ST136 (green) and CC151 (blue). Expression was determined relative to uninfected cells. Data represents mean log_2_-transformed, normalised expression of 3 individual strains ± SEM. Means which share a superscript are not significantly different.

All strains of *S*. *aureus* induced *CCL20*, *IL-1β*, *IL-6*, *IL-8* and *TNFα* expression in the MAC-T bovine mammary epithelial cell line (Supplementary File). Expression of *TLR2* was not detectable, despite verification that the hydrolysis probe assay could detect the transcript. Expression peaked at 6–12 hours post-infection (hpi) and either remained elevated or declined. There were no significant differences between *S*. *aureus* strains in their ability to induce an immune response from MAC-T cells at 1 hpi; however, for all other time points there were significant differences between strains in immune gene induction (Supplementary File). Strains belonging to the same lineage displayed remarkably similar patterns of immune gene induction with the exception of CC71, strains of which were more variable. Strains from CC97 were the strongest inducers resulting in a rapid increase in expression. The largest incitement of inflammatory signalling was mediated by CC97 strain, MOK028, which induced a >7000-fold increase in *CCL20* expression at 6 hpi. Moreover, both of the next most highly inducing strains also belonged to CC97. By contrast, ST136 and CC151 strains were relatively poor inducers with CC151 strains also displaying delayed immune gene induction for all genes except *IL-6* (Supplementary File). At 1 hpi there were no significant differences between lineages in immune stimulating properties but differences were evident at all other time points; CC97 was the highest immune gene inducer and ST136 and CC151 the poorest (Fig. 1A–E).

**Figure 2:**
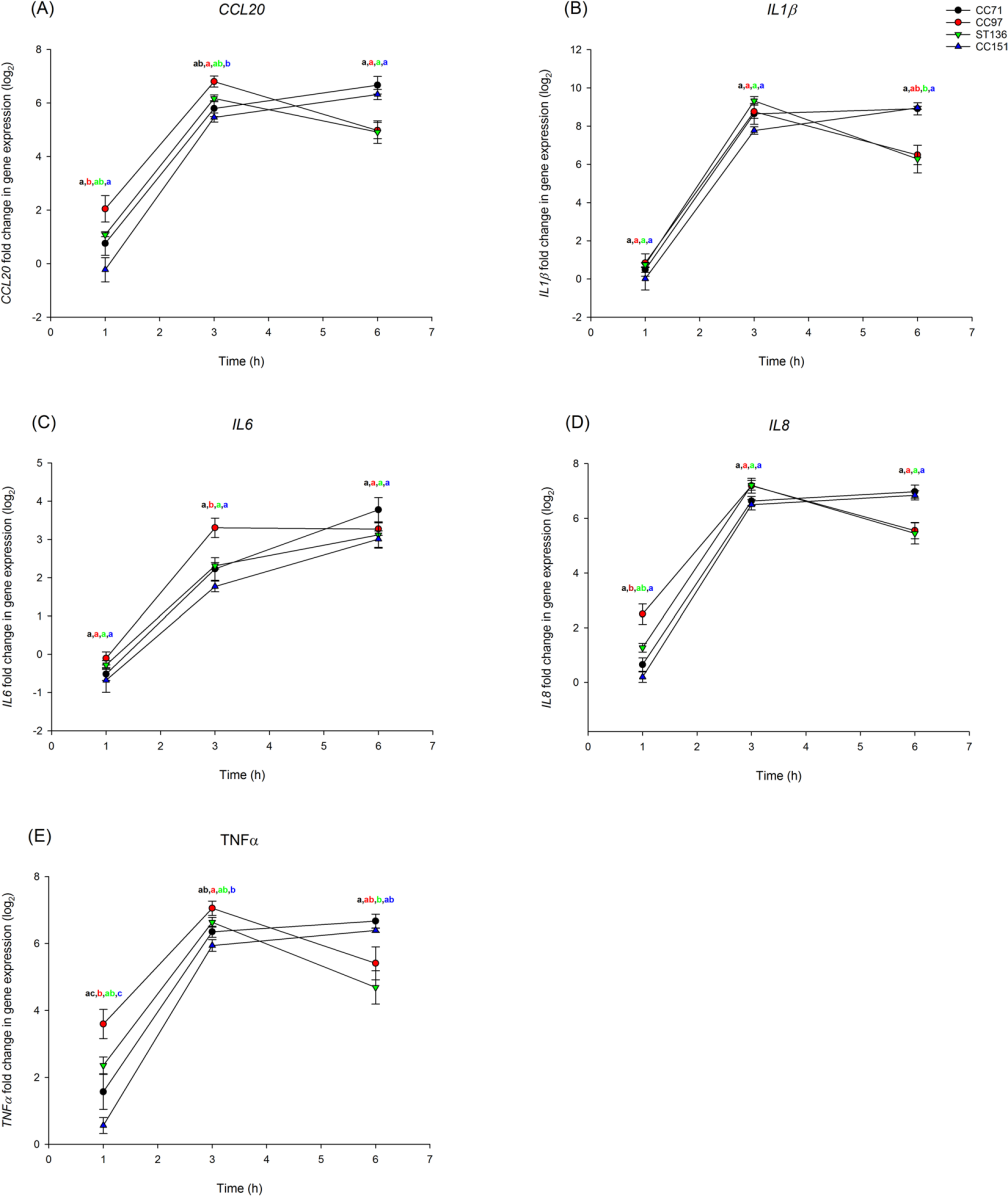
Gene expression in primary bovine mammary epithelial cells in response to infection with *S*. *aureus* from bovine-adapted lineages. Expression of (**A**) *CCL20*, (**B**) *IL1β*, (**C**) *IL6*, (**D**) *IL8* and (**E**) *TNFα* over time in primary bMECs infected with strains from *S*. *aureus* lineages CC71 (black), CC97 (red), ST136 (green) and CC151 (blue). Expression was determined relative to uninfected cells. Data represents mean log_2_-transformed, normalised expression of 3 individual strains ± SEM. Means which share a superscript are not significantly different.

Some differences were evident in the transcriptional response of pbMECs to infection with *S*. *aureus* strains but surprisingly these were much less pronounced than for MAC-T cells. Each of the tested genes was up-regulated, with some significant differences between strains in *TNFα* expression; these differences generally involved strains belonging to CC97 inducing gene expression more highly than those belonging to CC151 (Supplementary File). Again, considering lineages, a trend similar to that seen for MAC-T cells was evident; CC97 induced an earlier and higher pro-inflammatory response than other lineages (Fig. 2).

### Secretion of cytokines and chemokines differs by lineage of infecting strain

**Figure 3:**
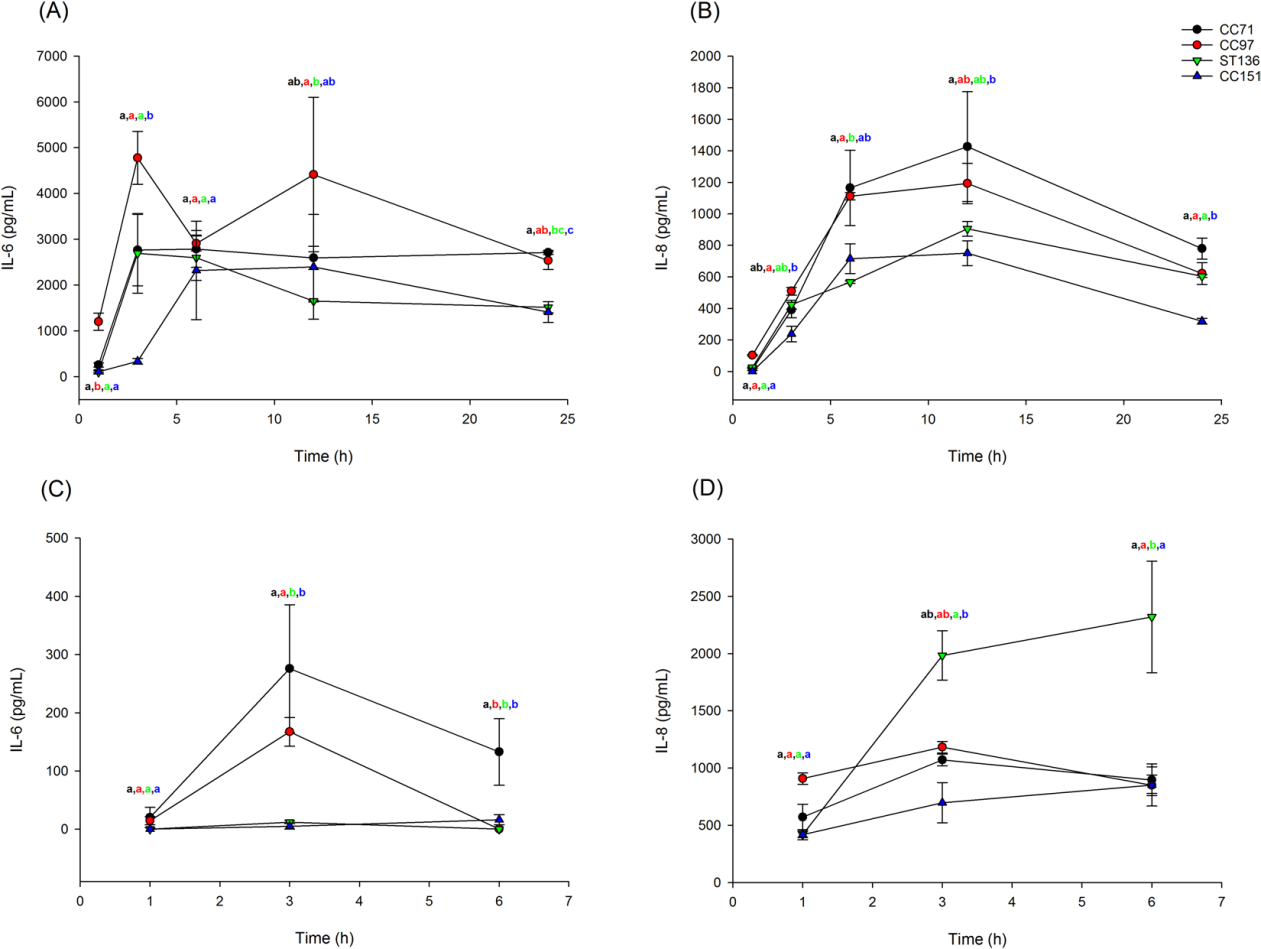
Secretion of IL-6 and IL-8 by bovine mammary epithelial cells in response to infection with *S*. *aureus* from bovine-adapted lineages. Secretion of IL-6 (**A**,**C**) and IL-8 (**B**,**D**) from MAC-T cells (**A**,**B**), or primary bMEC (**C**,**D**) over time in response to infection with strains from *S*. *aureus* lineages CC71 (black), CC97 (red), ST136 (green) and CC151 (blue). Data represent mean ± SEM of three strains per lineage. Means which share a superscript are not significantly different.

For each strain, the quantity of IL-6 and IL-8 secreted by epithelial cells during infection was determined. Uninfected MAC-T cells produced low levels of IL-6 (<110 pg/ml) at all time points except 24 h and low levels of IL-8 (<100 pg/ml) at all time points. Infection with all strains of *S*. *aureus* induced both IL-6 and IL-8 secretion from MAC-T cells (Supplementary File). Per lineage, CC97 induced significantly more IL-6 by 1 hpi than all other lineages and at 3 hpi all lineages induced more IL-6 than CC151. At 24 hpi CC97 and CC71 induced more IL-6 than CC151 (Fig. 3A). By 3 hpi CC97 also induced significantly more IL-8 than CC151 while at 24 hpi, all lineages had induced more IL-8 than CC151 (Fig. 3B).

Uninfected pbMECs did not secrete IL-6 at any time point and secreted low levels of IL-8. Infected cells induced expression of both IL-6 and IL-8 although expression of IL-6 was induced to a much lower level in pbMECs than in MAC-T cells (Supplementary File). There were many significant differences between strains in ability to induce IL-6 secretion while the only significant difference for IL-8 was that MOK076 induced significantly more IL-8 than MOK023 and MOK124 at 6 hpi (Supplementary File). At 3 hpi, CC71 and CC97 induced significantly more IL-6 than CC151 or ST136, while at 6 hpi CC71 induced more IL-6 than the other 3 lineages (Fig. 3C). For IL-8, ST136 induced secretion more highly than CC151 at 3 hpi and more highly than all lineages at 6 hpi (Fig. 3D).

### *S*. *aureus* lineages induce bMECs to produce chemotactic gradients of differing potency

**Figure 4:**
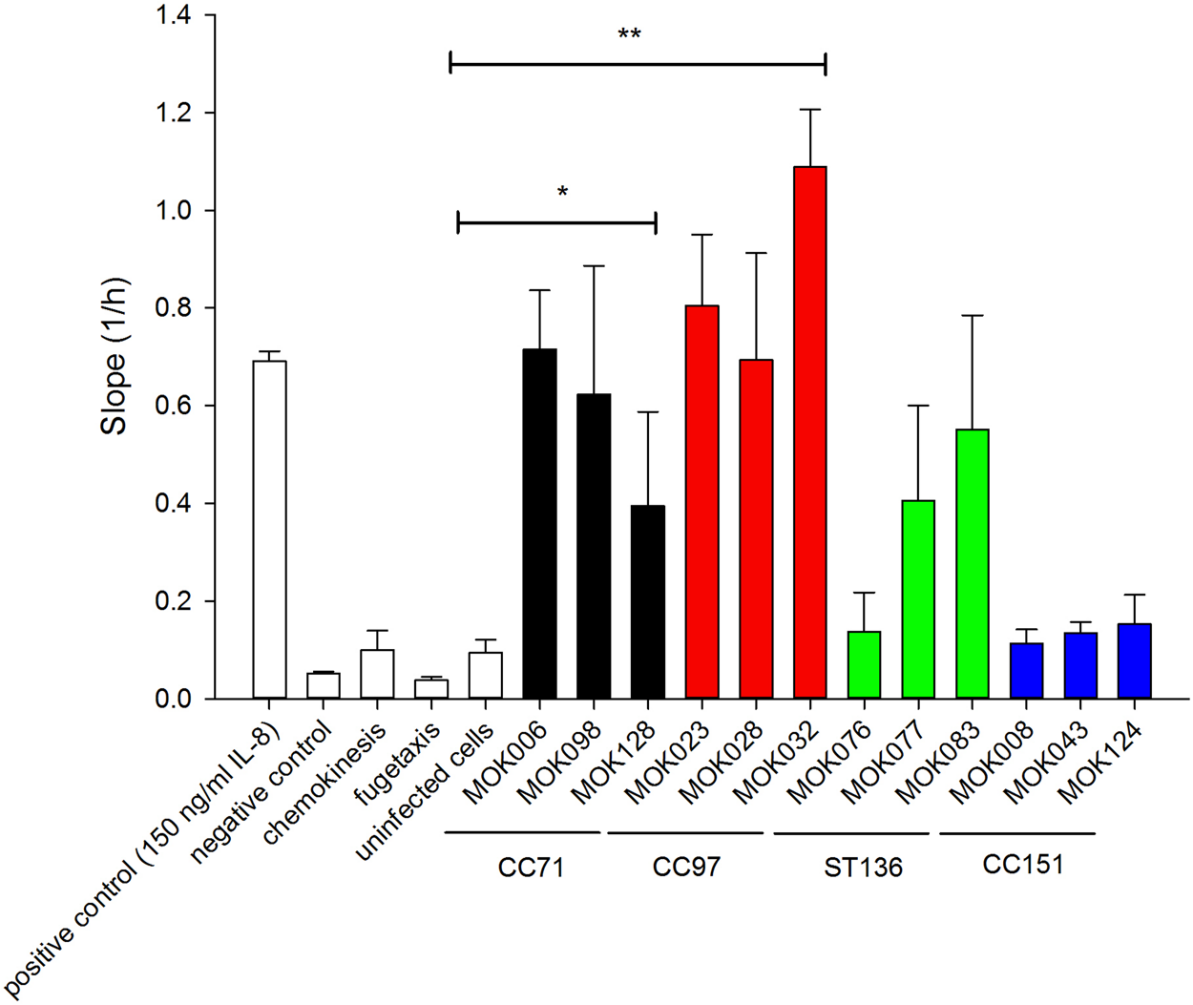
Ability of conditioned media from MAC-T cells infected for 24 h with each strain of *S*. *aureus* to attract neutrophils. Chemotaxis was measured in real time in a two-chamber cell migration plate by cell impedance on an xCELLigence system (Roche). Positive control included 150 ng/ml IL-8 in the lower chamber; negative control included no IL-8; chemokinesis control included 150 ng/ml IL-8 in both upper and lower chambers; fugetaxis control included 150 ng/ml IL-8 in the upper chamber while uninfected controls included conditioned media from uninfected MAC-T cells in the lower chamber. Data represent the mean rate of migration ± SEM, from triplicate experiments.

As individual strains, particularly those belonging to CC97, appeared to induce expression of pro-inflammatory mediators more highly than other strains, we sought to establish if this determined the ability of infected bMEC to attract immune cells. Conditioned media from MAC-T cells infected for 24 h with each strain of *S*. *aureus* were used as neutrophil chemoattractants in a real-time cell migration assay. All strains belonging to CC97 in addition to strains MOK006 and MOK098 (CC71) and MOK083 (ST136) were able to drive a migratory response that was significantly greater than conditioned medium from uninfected cells. Conditioned media from bMEC infected with CC151 strains displayed a striking lack of ability to stimulate neutrophil chemotaxis (Fig. 4). For the lineages, conditioned media from CC71 and CC97 resulted in significant chemotaxis (P = 0.02 and P = 0.006, respectively) while conditioned media from ST136 and CC151 did not (P = 0.09 and 0.82, respectively).

### Ability of *S*. *aureus* strains to survive killing by bovine granulocytes

**Figure 5:**
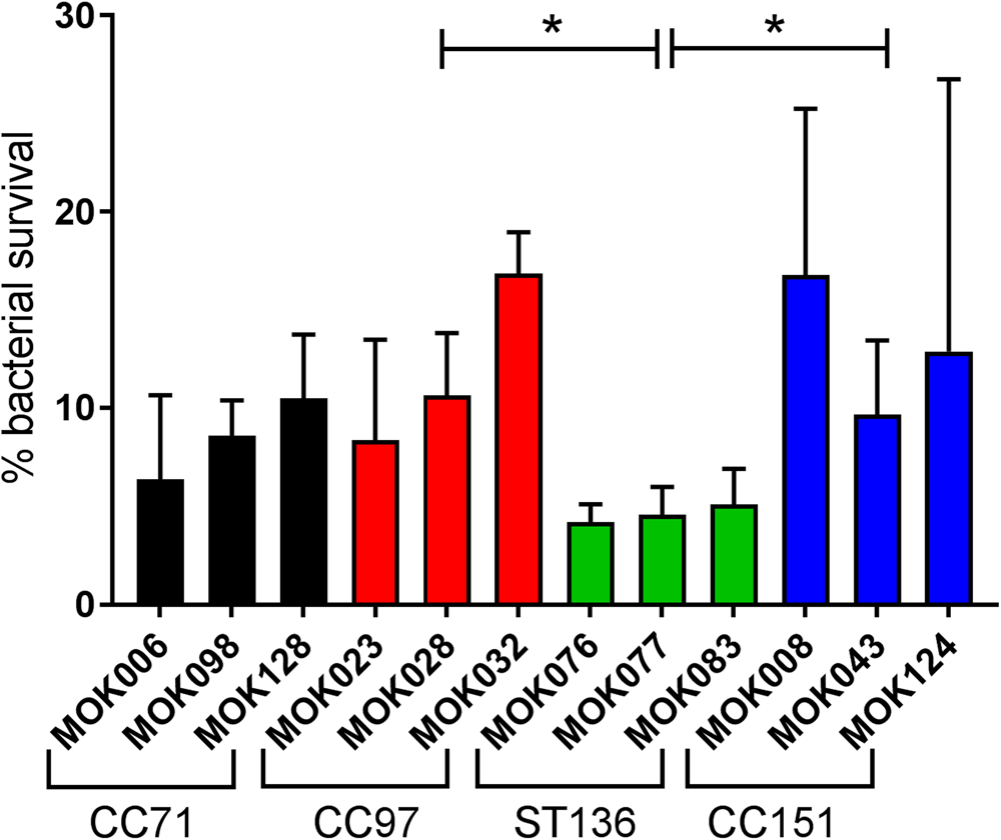
Survival of *S*. *aureus* incubated with bovine granulocytes. Percentage of each strain of *S*. *aureus* that survived 2 hours of co-culture with bovine granulocytes compared to cells incubated without granulocytes. Data represent the mean ± SD from triplicate experiments.

Each of the *S*. *aureus* strains was highly susceptible to the antimicrobial activity of bovine granulocytes following 2 hours of co-culture; survival ranged from 4.2% (MOK076; ST136) to 16.9% (MOK032; CC97) (Fig. 5). There were no significant differences between strains in ability to survive killing by bovine granulocytes (P = 0.1). There were, however, significant differences in survival between lineages, strains belonging to ST136 had significantly poorer survival than CC97 and CC151 (P < 0.05).

### Acute infection causes a lineage-dependent effect on cell behaviour

**Figure 6:**
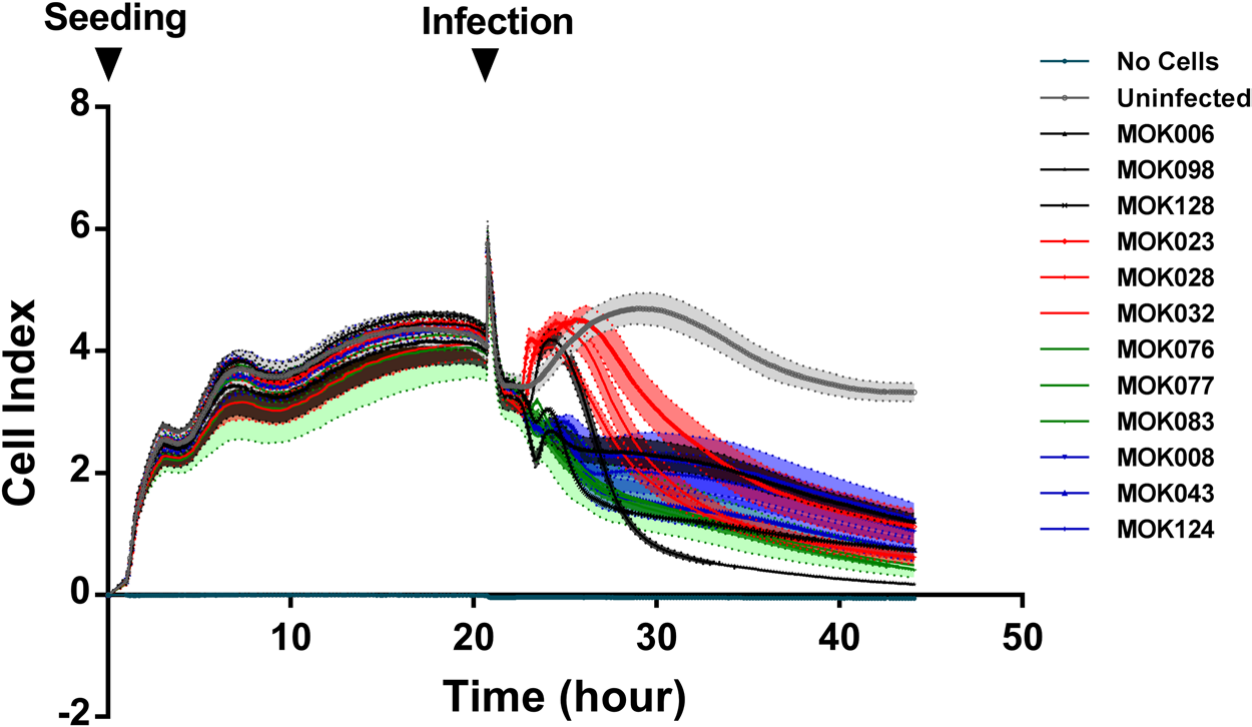
Real-time measurement of adhesion, morphology and detachment of MAC-T cells pre- and post-infection with each of 12 strains of *S*. *aureus*. Cells were cultured to confluence in wells of an E-plate (Roche) over 20 h before addition of each *S*. *aureus* strain at an MOI of 10 (arrow indicates time of infection). Cell index, the relative change in electrical impedence and hence a measure of the strength of cell adhesion, was monitored in real-time for the following 24 hours post-infection using an xCELLigence system (Roche). Data represent mean cell index ± SD over time (N = 3 per strain).

In order to determine whether the strains differentially affected cell viability, monolayer cultures of MAC-T cells were established in E-plates. Cells were confluent by 20 h of culture. For this cell type, confluence corresponded to a cell index (CI) of ~4. A short-lived disruption in impedance was evident due to plate removal for replacement of media and inoculation of bacteria. For uninfected cells, CI remained steady over the remainder of the experiment. Infection with *S*. *aureus* caused a decline in cell index over time, indicating detachment of non-viable cells from the electrodes. Individual strains affected MAC-T cells differently; infection with all strains belonging to CC97 as well as MOK098 (CC71) resulted in a transient rise in cell index followed by a rapid decline while infection with the remaining strains resulted in a prolonged gradual decline in cell index (Fig. 6).

### *S*. *aureus* strains show differences in their ability to internalise within epithelial cells

**Figure 7:**
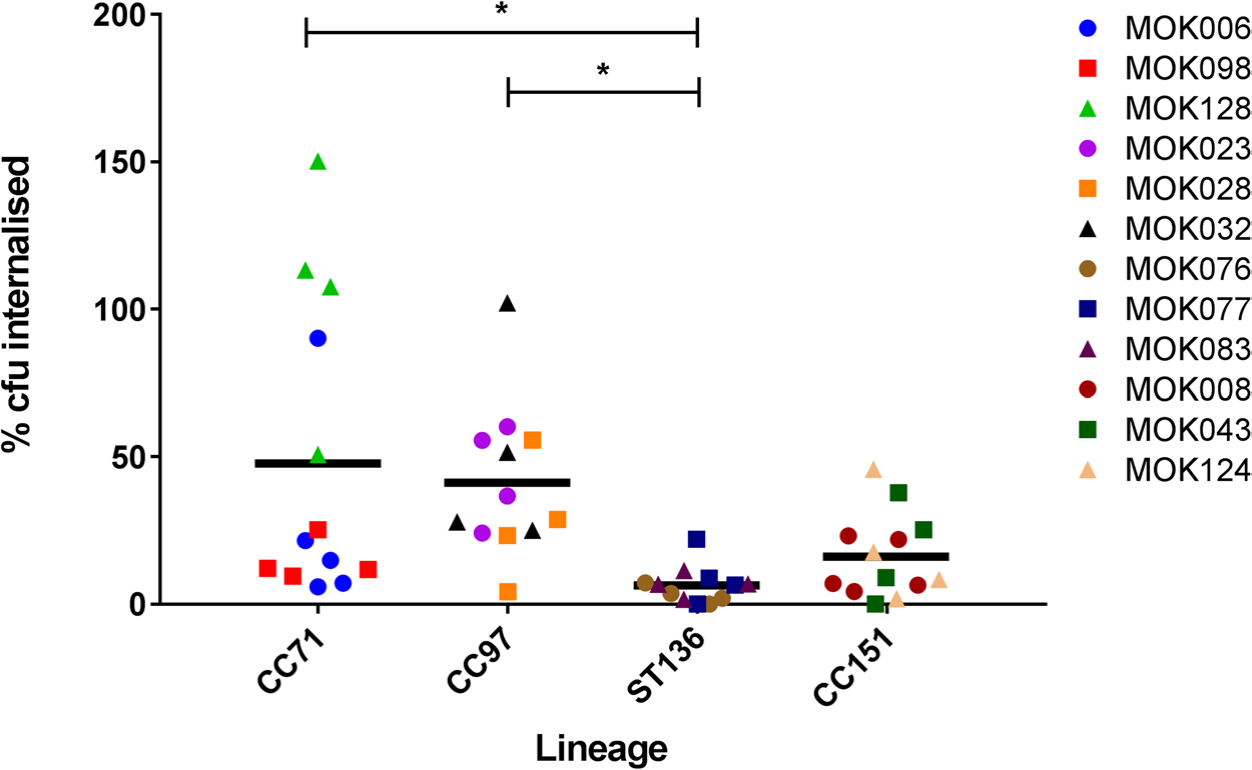
Percentage of each strain of *S*. *aureus* that internalised into MAC-T cells after 2 hours of co-incubation. Percentage internalisation was determined relative to bacterial proliferation in the absence of cells. Data represent mean ± SD, N = 4.

Invasion and persistence is a trait common to multiple strains of *S*. *aureus*. Hence, we determined whether the strains differed in their ability to invade MAC-T cells. Strain MOK128 (CC71) was significantly more effective at internalising into MAC-T cells than all strains belonging to ST136, while strains MOK023 and MOK032 internalised to a higher extent than MOK076 (P < 0.05) (Fig. 7). There were no significant differences between any other strains. Comparing between lineages, CC97 and CC71 strains internalised to a significantly greater extent than ST136 strains (P < 0.05).

Differences between the lineages in ability to elicit the production of chemotactic factors from bMEC, ability to resist killing by bovine neutrophils and ability to internalise into bMEC are summarised in Table 1.

**Table 1:** Summary characteristics of bovine-adapted *S*. *aureus* lineages.

Lineage	Capsule type	bMEC Internalisation	Neutrophil Survival	Recruitment of neutrophils by bMEC conditioned media
CC71	*cap8*	49.4%^a^	8.5%^ab^	0.55*
CC97	*cap5*	41.3%^a^	12.0%^a^	0.80*
ST136	*cap5*	6.4%^b^	4.6%^b^	0.36
CC151	*cap8*	16.4%^ab^	13.1%^a^	0.12

*Significantly different from uninfected control.

Means which share a superscript are not significantly different.

## Discussion

*Staphylococcus aureus* mastitis is a global problem with substantial economic costs and negative impact on animal health and welfare. Current mastitis control methods such as disease detection, culling of persistently infected animals and genetic selection for mastitis resistance are primarily based on the host response to infection, determined by milk somatic cell count (SCC). Therefore the ability of *S*. *aureus* strains to interact in significantly different ways with the host immune system may impact diagnosis, treatment and control of disease. Mammary epithelial cells are one of the major host cell types responsible for initial detection of infecting *S*. *aureus* and the severity and outcome of infection is dependent on the ability of this cell type to signal to professional immune cells in a timely manner, initiating rapid and substantial neutrophil recruitment^17^. In this study we characterise the bMEC response to infection for 24 h with live *S*. *aureus* from different bovine-adapted lineages for the first time. We demonstrate *in vitro* an interaction between bMEC response and the genotype of *S*. *aureus* and demonstrate that this has downstream consequences for immune signalling.

Each of the examined *S*. *aureus* strains induced expression of immune signalling cytokines and chemokines in the bMEC cell line MAC-T. Substantial up-regulation occurred within 3 hpi, suggesting a robust, acute response by the cells to *S*. *aureus*. CC97 strains proved highly immunogenic, while strains from other lineages, most notably CC151 and ST136, were much less immune-stimulatory. Notably, gene induction was similar among strains that were of a common lineage, but differed significantly between those of different lineages. Strains of CC151 also appeared to display a somewhat delayed immune gene induction compared to other strains. This suggests that some lineages are more immunogenic than others and/or some may be immunosuppressive. A relative failure of the immune system to properly detect *S*. *aureus* compared to *E*. *coli* has been previously reported^7,8,18–21^; albeit in studies comparing heat-killed isolates, which may not fully reflect the dynamic interaction between host cells and live bacteria. These studies demonstrated that *S*. *aureus* was weakly agonistic for TLR2 and failed to induce NF-κB and its downstream effectors in pbMECs^7,8,19^. It was recently shown *in vivo* that this failure underpins the differential presentation of mastitis caused by these species with *S*. *aureus* being actively immune evasive through NF-κB suppression. In the present study, expression of *TLR2* was not detected in response to any strain and so other effectors may be responsible for the observed differences in immunogenicity. Lack of TLR2 stimulation in human cells transfected with bovine TLR2 has been previously observed in response to other Gram positive bacteria which cause IMI such as *S*. *uberis* and *S*. *agalactiae*^21^. The expression of capsular polysaccharide by *S*. *aureus* has also been reported to interfere with bacterial recognition via TLR2 possibly by masking TLR2-active lipoproteins^22^.

In support of our observations regarding gene expression, we assayed the secretion by the cells of IL-6 and IL-8. IL-6 is a cytokine required for Th17 cell differentiation, a cell type that releases IL-17 instigating a signalling cascade that results in recruitment and activation of neutrophils^23^. IL-8 is a key chemokine required for neutrophil recruitment and both cytokines have previously been shown to be central to the mammary epithelial response to *S*. *aureus* mastitis^8^. Infected MAC-T cells secreted IL-6 and IL-8 over 24 hours of infection to different extents depending on the infecting strain, in agreement with the differential gene expression of *IL-6* that we observed. Although both gene and protein expression assays generally found greatest cytokine production to be stimulated by CC97 or CC71 strains with a lesser pro-inflammatory response to CC151 strains, the concordance between mRNA expression and that of the cognate protein was not absolute. Such discrepancies are frequently reported and a variety of explanations are possible including mRNA stability, translational regulation and protein turnover. In this instance the reason for any discrepancies is unknown but importantly functional neutrophil chemotaxis assays confirmed that infection with strains from CC97 resulted in the strongest neutrophil recruitment while infection with strains from CC151 failed to result in neutrophil recruitment.

It has previously been shown for *S*. *aureus* strains that the magnitude of induction of *IL-1β*, *IL-8* and *TNFα* from bMEC varied according to strain^24^. In the aforementioned study, however, extracellular *S*. *aureus* were killed at 3 hpi and gene expression returned to basal levels by 24 hpi, while in our study gene expression remained elevated (MAC-T) or infection resulted in cell death (pbMEC)^24^. Elsewhere, heat-inactivated *S*. *aureus* strains were shown to elicit differing immune responses from pbMECs^15^, with the more pro-inflammatory strains belonging to lineages CC8, CC20 and CC97, and the less agonistic strains all belonging to CC151, thus supporting our finding of relatively limited immune signalling mediated by this lineage. These studies support our finding that different strains of *S*. *aureus* can induce immune gene expression to differing extents and that this difference is likely mediated through lineage-specific factors. In many studies, only single strains of *S*. *aureus* are utilised to investigate the response of the bovine mammary epithelium to challenge with *S*. *aureus*^8,18,19^. Hence, these studies lack information about the potential for differing strains of *S*. *aureus* to present with differing immunologic profiles in the mammary gland. Limiting the study of *S*. *aureus* mastitis to one or a few closely related strains may overlook the range of immunogenicity of which the species is capable.

Variation in the immune response of pbMEC to *S*. *aureus* strains was also evident, albeit with somewhat lesser magnitude of expression and more moderate differences between lineages. This was surprising as it has been previously reported that while MAC-T cells faithfully reproduce key aspects of the parental cell immune response, their response tends to be weaker^25^. It is not clear why, in our hands, the pbMEC response was not completely concordant with that of MAC-T but it may reflect individual variability between MEC donors, a loss of response of our cells due to freezing and passage or the fact that infection with live bacteria (compared to heat-killed in^25^), which led to death of the infected monolayers, affected gene expression. Alternatively, as MAC-T and pbMEC were each cultured in a cell-specific medium this may have impacted gene expression; the composition of culture medium has been previously reported to modulate gene expression in *S*. *aureus*-infected MEC^26^.

Differences between the lineages in host cell interactions were further evident in their effect on cell morphology and viability. Infection of MAC-T cells with all CC97 strains, as well as with one CC71 strain, resulted in a transient increase in cell index (CI) during the initial phase post-infection. This was in contrast to the immediate decline in CI caused by the remaining *S*. *aureus* strains. There were also slight differences between strains in the rate of detachment, and some cells appeared detached by 24hpi. This indicates that the 24 hpi cytokine induction and secretion are caused by a smaller subset of cells, and also that some differences observed at this time point are caused by cell death processes. However, further studies are necessary to examine the viability of bMEC when infected with the different strains. The transient increase in CI most likely reflects a change in cellular morphology, such as cellular swelling mediated by pyroptosis pursuant to inflammatory signalling, or necrosis, in response to a factor expressed by these strains^27,28^.

*S*. *aureus* can internalise within host cells, including epithelial cells and phagocytes^29–33^, and can be found within epithelial cells and macrophages isolated from cows experiencing IMI^34^, or in the interstitial tissue particularly during chronic infection^35^. Here they may evade the immune system, establishing a reservoir from which to re-establish infection. A *S*. *aureus* strain with a reduced capacity for mammary epithelial cell internalization *in vitro* was also shown to have delayed mammary epithelial cell internalisation *in vivo* in a murine mastitis model^36^. In the present study a high degree of variability in the extent of the strains’ internalisation was evident not only between strains, but also for the strains themselves. With the exception that ST136 strains were consistently poorly internalised, within strain and lineage variation was large. This is consistent with a previous report of large variation in the ability of bovine *S*. *aureus* isolates to invade MAC-T cells^37^. Nevertheless, we observed the minimum internalisation of our strains to be 4.3 ± 2.6%, in the case of the ST136 strain MOK076. By contrast, some studies have reported invasion by geographically diverse mastitis isolates of *S*. *aureus* into MAC-T or pbMEC cells below 0.01% of initial inoculum, even when a high bacterial titre (10^9^ cfu) was used^33,38^. It has been shown that the well-characterised bovine isolates, RF122 (CC151) and Newbould 305 (CC97), internalise into MAC-T cells up to ~1% and ~15% of their respective initial titres when added in a 100:1 MOI to the cells^39^. Thus, our findings support the previously reported differences in the proficiency of various strains to internalise into bMECs.

Neutrophils are the predominant infiltrating cells during the response to *S*. *aureus* intramammary infection, accounting for over 80% of milk somatic cells during acute infection^40^ and 66% of somatic cells in sub-clinical infection^41^, remaining dominant during persistent infection^42^. Importantly, as well as demonstrating that infection with CC97 strains resulted in upregulation of immune gene and protein expression while CC151 strains caused a delayed response of lower magnitude, we also demonstrated that this upregulation had a functional effect on neutrophil recruitment. Hence, the infecting strain determined the ability of bMEC to signal to professional immune cells. A delayed influx of neutrophils into the mammary gland negatively affects infection resolution, allowing the infection to become established which can lead to invasive disease. Therefore the delayed and muted immune response initiated by CC151 strains may be a strategy to allow their establishment in the intramammary environment. A number of epidemiological studies have examined the relationship between *S*. *aureus* genotype and infection characteristics although a more limited number have characterised the isolates by clonal complex; among these studies it has been reported that *S*. *aureus* transmission and cure depend on lineage^43–46^. The majority of *in vivo S*. *aureus*-challenge IMI studies have been conducted with strains belonging to ST8 (CC8), ST133 (CC133) or ST115 (CC97; the laboratory strain Newbould 305)^35,47–49^ and in some instances where a local isolate has been used the infecting strain genotype has been omitted^50,51^. The relevance of using Newbould 305 to study *S*. *aureus*-mediated bovine mastitis has been questioned on the basis of its mild induction of host response and the limited distribution of ST115 strains^52^. Interestingly, although it has been reported that experimental infection with *S*. *aureus* strain RF122 (CC151) causes a severe mastitis while Newbould 305 (CC97) causes mild mastitis^13^, RF122 has not been widely studied *in vivo*. A pilot study by Wilson^53^ described infection of 3 cows with RF122. In all instances infection was established and acute clinical mastitis was observed with an inoculum as low as 2 cfu. A further study by the same group reported clinical mastitis in 3 out of 4 cows infected with RF122^54^.

The ability of neutrophils to phagocytose and kill invading *S*. *aureus* is vital for infection resolution. In turn, some strains of *S*. *aureus* are capable of producing pore-forming leukocidins and high expression of the bovine LukMFʹ leukocidin has been associated with severe mastitis^55^. Carriage of the *lukMFʹ* genes is lineage-dependent and among our strains only CC151 strains encoded both genes required to form the two-component toxin^16^. Lineages CC97 and CC151 were more resistant to killing than ST136 but there were no significant differences between individual strains, suggesting that expression of *lukMFʹ* may not have been switched on under the assay conditions. Neutrophils recruited to the mammary gland show an immature phenotype, and their enzymatic activity is perhaps 10% that of blood-derived counterparts^56^ with milk neutrophils showing reduced capacity to kill *S*. *aureus* compared to blood derived neutrophils^57^. As such, the bacteria may have greater opportunity to survive in the udder, where strain-specific differences may be evident.

In the present study, the marked difference in the immunogenic character of the strains may be due to their lineage-dependent carriage of virulence traits including adhesins and toxins^16^. The CC97 strains harbour multiple Microbial Surface Components Recognising Adhesive Matrix Molecules (MSCRAMMs) and subsequently may be invasive and capable of producing persistent subclinical mastitis *in vivo*. Conversely, the CC151 strains do not encode a number of MSCRAMMs including fibronectin binding protein B (*fnbB*) and may therefore fail to readily adhere and internalise^58^. In this context, the expression of LukMFʹ by CC151 strains may dispose them to cause acute clinical mastitis^59,60^. Further to this, the CC151 strains are *agr* type II and *cap8*, a genetic profile suggested to be adapted to the extracellular niche^37^. CC151 isolates are also poor biofilm formers relative to CC97 isolates^14^. This could bring CC151 strains in direct contact with the host immune response; biofilm formation being associated with immune evasion and persistent intramammary infection^11^. Differences between strains within a lineage were expected, as the accessory genome of each differs to some extent. The redundancy of MSCRAMMs and toxins within the global *S*. *aureus* pangenome perhaps explains why no strain was entirely deficient in either invasion of MAC-T cells or induction of immune response genes. While it is compelling to consider that, in order to persist sub-clinically in the udder, *S*. *aureus* must be minimally immunogenic, this need not be the case if the bacteria survive intracellularly.

Greater variability was observed among CC71 strains than among strains of the other lineages in ability to internalise, elicit an immune response and stimulate neutrophil chemotaxis. Some CC71 strains also behaved similarly to CC97 strains in their ability to elicit expression of chemotactic factors and their effect on bMEC morphology. CC71 is a recently defined lineage which evolved from CC97 by multiple recombination events in an approximately 300 kb region spanning the origin of replication^61,62^. In this study, strains belonging to CC71 were of two different sequence types, ST71 and ST3173, which may explain in part the differences in host cell interaction observed. A previous study also demonstrated phenotypic variability between ST71 strains^62^. Therefore diversity of strains within the CC71 lineage may be greater than within other lineages, which warrants further examination.

Using a panel of *S*. *aureus* strains of known bovine-associated lineage, we have shown that strain interaction with bovine mammary epithelial cells and neutrophils varies according to bacterial genotype. The differences in bMEC interaction and bacterial survival indicate that each *S*. *aureus* lineage has a unique set of characteristics that may determine the outcome of infection *in vivo*. Further studies are required on the differences between strains in their ability to cause intramammary infection in cattle. This work also emphasises that study of single strains, even model-specific isolates, can misrepresent the breadth of possible outcomes of infection across a bacterial species. Associating the specificity in virulence with lineage and strain in future studies would enable more effective comparison of general trends in *S*. *aureus* virulence.

## Methods

### Bacteriological Culture

The strains were originally isolated each from a different cow presenting with clinical mastitis; in total 6 farms are represented. Isolation and characterisation of the strains has been previously described^4,16^. Bacteria were cultured until stationary phase in tryptic soy broth (TSB; BD Bioscience) at 37 °C, 220 rpm. Cultures were suspended in phosphate buffered saline (PBS; Gibco) to an optical density (OD_600nm_) of 1 prior to dilution in appropriate culture medium. Colony forming units were enumerated by manual count following serial dilution in PBS, spreading onto tryptic soy agar and overnight incubation at 37 °C. The *S*. *aureus* strains used in the study are described in Table 2. The genome of each strain has been sequenced and deposited in the NCBI Sequence Read Archive; accession numbers are listed in Table 2.

**Table 2:** Lineage and Sequence Type of *S*. *aureus* strains used during this study.

Strain^†^	Lineage	Sequence Type	Genome Accession Number
MOK006	CC71	ST3173	SRS2841719
MOK098	CC71	ST71	SRS2841716
MOK128	CC71	ST71	SRS2841712
MOK023	CC97	ST3170	SRS775827
MOK028	CC97	ST3221	SRS2841721
MOK032	CC97	ST3170	SRS775824
MOK076	ST136	ST136	SRS2841714
MOK077	ST136	ST136	SRS2841715
MOK083	ST136	ST136	SRS2841717
MOK008	CC151	ST151	SRS2841718
MOK043	CC151	ST151	SRS2841720
MOK124	CC151	ST151	SRS2841713

^†^Full characterisation of strains in^16^.

### Cell Culture

MAC-T cells, a bovine mammary epithelial cell line derived from a lactating Holstein cow^63^ were recovered from −150 °C (passage 9–10) and cultured in Dulbeccos’ Modified Eagle medium (DMEM; Sigma) with 10% foetal bovine serum (FBS; Sigma) at 37 °C, 5% CO_2_. Primary bovine mammary epithelial cells (pbMECs; AvantiCell Ltd) were derived from the udder parenchyma of a cow during the third trimester of pregnancy. Primary cells were cultured as described previously^64^. All cells were used within six passages following revival from storage at −150 °C.

### Cell Infections and RNA Extraction

MAC-T cells were seeded into 12-well plates at 2 × 10^5^ cells/well in 1 ml DMEM, 10% FBS. Primary cells were seeded into EHS matrix-coated 12-well plates at 8 × 10^5^ cells/well in 1 ml of attachment-differentiation medium, replaced with serum-free lactogenic medium after 24 h. Cells were incubated at 37 °C, 5% CO_2_ until confluent. Monolayers were washed twice with pre-warmed infection medium (DMEM with 1% FBS for MAC-T; serum-free lactogenic medium for pbMECs) before inoculation with bacteria at a multiplicity of infection (MOI) of 10. Uninoculated wells served as uninfected controls. Following 1, 3, 6, 12 or 24 h of incubation at 37 °C, 5% CO_2_, medium was removed, clarified by centrifugation and stored at −20 °C. The viability of *S*. *aureus* infected MAC-T at 24 h post-infection was 80–90% using trypan blue exclusion and annexin V/PI staining while pbMEC were non-viable by 12 h post-infection. Wells were washed with 1 ml pre-warmed PBS and RNA was extracted using RNeasy kit (Qiagen) and stored at −80 °C.

### Gene expression analysis

RNA was treated to remove residual DNA using the Turbo DNA-free kit (Ambion) and quantified using the RNA broad range assay kit (Invitrogen). RNA quality was verified with a 2100 bioanalyser (Agilent). Synthesis of cDNA was conducted on MAC-T samples having RIN values exceeding 7 (mean 9.6) and on primary cell samples having RIN values exceeding 5.4 (mean 8.4). RNA from each sample (500 ng) was reverse transcribed using the Superscript VILO kit (Invitrogen).

For each sample, cDNA (1 µl) was added to duplicate wells with 7 µl of DNase-free water, 10 µl of Taqman Fast Advanced master mix (Applied Biosystems) and 1 µl of each of 2 Taqman gene expression assays (Applied Biosystems) (Table 3). Lack of interference between assays was confirmed experimentally. Samples were incubated at 50 °C for 2 min followed by 95 °C for 20 s then cycled 40 times at 95 °C for 3 s and 60 °C for 30 s in a 7500 Fast thermocycler (Applied Biosystems).

**Table 3:** Taqman assays used for analysis of gene expression in *S*. *aureus*-infected bovine mammary epithelial cells.

Gene	Gene Accession	Taqman^®^ Assay ID	Fluorophore	Amplicon Length
*IL-6*	NM_173923.2	Bt03211903_m1	FAM	69
*CCL20*	NM_174263.2	Bt03223359_m1	FAM	73
*TLR2*	NM_174197.2	Bt03223212_m1	VIC	109
*IL-8*	NM_173925.2	Bt03211907_g1	FAM	105
*IL-1β*	NM_174093.1	Bt03212744_m1	FAM	69
*TNFα*	NM_173966.3	Bt03259155_g1	VIC	66
*ACTB*	NM_173979.3	Bt03279175_g1	VIC	144
*RPS24*	NM_001025339.2	Bt03220533_g1	VIC	87

Target gene Cq values were normalised against the reference genes *ACTB* and *RPS24*, selected from 9 candidate genes whose stable expression in both MAC-T cells and pbMECs that were infected with *S*. *aureus* was evaluated using GeNorm, NormFinder and BestKeeper. The efficiency of all PCR reactions was between 98–102% (data not shown). Fold change in gene expression level was calculated by comparing to Cq values from uninfected cells at the same time point.

### Quantification of secreted proteins by ELISA

For quantification of cytokine secretion, 100 µl of supernatants recovered post-infection was assayed by sandwich ELISA for the presence of IL-6 (ThermoFisher) and IL-8^65^, per manufacturers’ instructions. All samples were assayed in duplicate and values below the limit of detection (39 pg/mL for IL-6 and 31 pg/mL for IL-8) were set to zero.

### Neutrophil-mediated killing of bacteria

Peripheral blood was collected from four disease-free cattle. Serum was collected from the same four animals and pooled. Erythrocytes were lysed by suspension of blood for 10 min in four volumes of high-yield lyse solution (Invitrogen) at ambient temperature. Leukocytes were isolated by centrifugation at 300 × *g* for 10 min and washed in sterile PBS. Granulocytes were purified by magnetic-activated cell sorting, using mouse anti-bovine granulocyte IgG_1_ (clone CH138A Kingfisher Biotech Inc) and magnetic anti-mouse IgG_1_ microbeads (Miltenyi Biotec), following the manufacturers’ protocol (Miltenyi). Estimation of purity and enumeration of granulocytes was performed on an Attune flow cytometer (Applied Biosystems). Granulocytes were centrifuged for 10 min at 300 × *g* and re-suspended to 1 × 10^7^ cells/ml in RPMI.

Fresh bacterial colonies were suspended in sterile PBS to an OD_600nm_ of 0.45 and enumerated as described above. Each strain (7 × 10^6^ cfu in 50 µl) was suspended in the presence or absence of 2.5 × 10^6^ granulocytes (MOI ~3) to a total volume of 500 µl including 10% autologous bovine serum and incubated at 37 °C for 2 h, rotating end-over-end. Ultrapure water was then added directly to lyse granulocytes and suspensions were serially diluted in sterile PBS. Dilutions (50 µl) were spread onto triplicate TSA plates for enumeration of bacteria. The experiment was conducted in triplicate for each strain.

### Real-time cell adhesion and morphology during infection

Wells of a 16-well E-plate (Roche Diagnostics GmBH) were equilibrated for 30 min at ambient temperature with 100 µl DMEM before adding 1 × 10^4^ MAC-T cells/well in an additional 100 µl DMEM. One well per plate received DMEM only to control for background impedance. Cells were allowed to settle for 30 min before plates were inserted into an xCELLigence RTCA DP Analyser (ACEA Biosciences Inc.) at 37 °C, 5% CO_2_. Impedance was measured for 20 h. E-plate wells were confirmed visually to be confluent with cells. Medium was removed and each strain was added to a discrete well at an MOI of 10 in 200 µl DMEM. Bacteria were allowed to settle for 30 min and E-plates were returned to the instrument at 37 °C, 5% CO_2_ for a further 24 h. The experiment was conducted in triplicate for each strain.

### Cell Invasion Assay

MAC-T cells were infected for 2 h with *S*. *aureus* as described above. Wells were washed with PBS and 1 ml of 100 µg/ml gentamicin in DMEM plus 1% FBS was added. Plates were incubated for a further 1 h then washed with PBS. Cells were lysed for 10 min in 1 ml 1% Triton X-100, 0.1% SDS, followed by repeated pipetting. Lysates were serially diluted in PBS and plated (50 µl) to enumerate internalised bacteria. The inoculum was similarly incubated for 2 h before dilution and plating. The experiment was conducted in quadruplicate for each strain.

### Chemotaxis Assay

The chemotaxis assay was carried out as described elsewhere^66^. Briefly, cell invasion and migration plates (CIM-Plate 16; Roche) were coated with 20 μg/ml fibronectin (Sigma) in PBS at 37 °C, 30 min, then washed twice with 50 μl PBS. The lower chambers were filled with 160 µl of supernatants from MAC-T cells infected with the relevant strain of *S*. *aureus* for 24 h. Upper chambers received 35 µl DMEM with 1% FBS. After 1 h at room temperature, background impedence was obtained at 37 °C, 5% CO_2_. To the top chamber of each well, 100 µl of neutrophils (1.2 × 10^7^ cells/ml in DMEM with 1% FBS) was added and impedence measured every 5 min for 7 h. Several controls were included: (i) positive control: neutrophils in upper chamber, 150 ng/ml bovine IL-8 (Kingfisher Biotech Inc) in lower chamber (ii) negative control: neutrophils in upper chamber, 0 ng/ml IL-8 in lower chamber (iii) chemokinesis: neutrophils in upper chamber, 150 ng/ml IL-8 in upper and lower chambers (iv) fugetaxis: neutrophils and 150 ng/ml IL-8 in upper chamber, medium only in lower chamber; (v) uninfected cells: neutrophils in upper chamber, medium from cells uninfected with *S*. *aureus* in lower chamber. The slope of the exponential part of the curve (1 h) was calculated using RTCA software 1.2.1.

### Statistical Analysis

Models were fitted and residuals checked to ensure the assumptions of the analysis (normality and constant variance) were met; log transformation was used as appropriate. Transformations were applied to ELISA measurements (sqrt(x)), internalisation measurements (log_10_(x + 1)) and chemotaxis measurements (log_10_(x*100 + 1). For between strain analyses, data were analysed using Proc GLIMMIX (SAS v9.3); for two-factor experiments, a random time statement with a residual qualifier and unstructured covariance matrix accounted for repeated measures over time. For the between lineage analysis, data were also analysed using Proc GLIMMIX, with strain as a random term. Tukeys’ *post-hoc* multiple comparison test or Dunnetts’ comparison to a control were applied as appropriate. P values < 0.05 were considered significant.

### Ethical approval

All procedures involving animals were conducted under ethical approval and experimental license from the Irish Health Products Regulatory Authority and performed according to European regulations regarding animal welfare and protection of animals used for experimental and other scientific purposes.

## Supplementary information

Supplementary File
