# Supplementary material for: The in vitro host cell immune response to bovine-adapted Staphylococcus aureus varies according to bacterial lineage
Source: Sci Rep. 2019 Apr 16;9:6134. doi: 10.1038/s41598-019-42424-2 (PMC6467978; doi:10.1038/s41598-019-42424-2)
Supplement: Supplementary file 1 — Supplementary File [file 41598_2019_42424_MOESM1_ESM.pdf]

Supplementary Information

The *in vitro* host cell immune response to bovine-adapted *Staphylococcus aureus* varies according to bacterial lineage

Mark P. Murphy[a*], Dagmara A. Niedziela[a,b], Finola C. Leonard[b], Orla M. Keane[a]

[a] Animal & Bioscience Department, Teagasc, Grange, Dunsany, Co. Meath, Ireland

[b] School of Veterinary Medicine, University College Dublin, Belfield, Ireland

This file includes Supplementary Information: Gene and protein expression in MAC-T and primary bovine mammary epithelial cells in response to infection with 12 strains of *S. aureus* belonging to bovine-adapted lineages CC71, CC97, ST136 and CC151 and significant differences between strains.

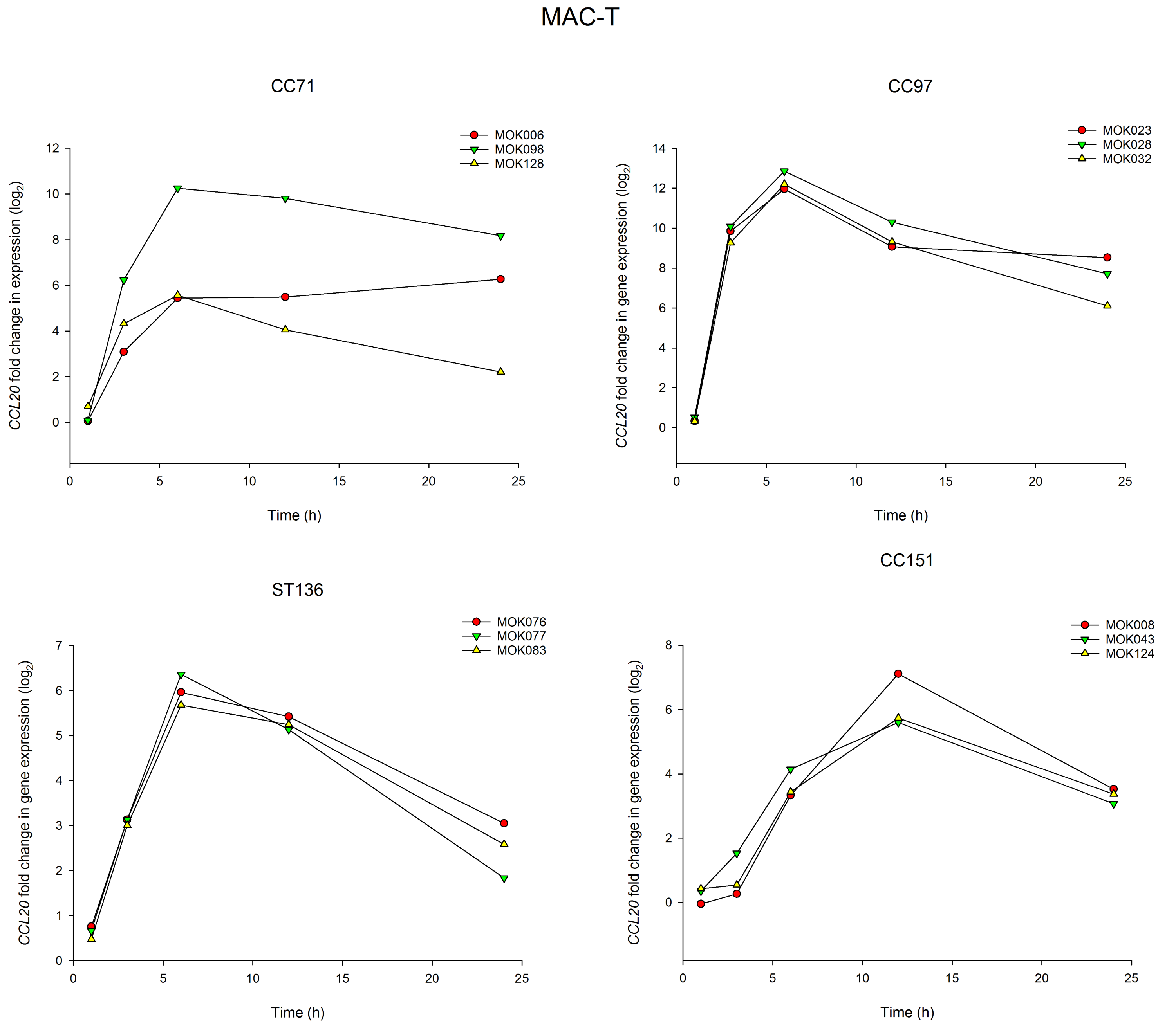
MAC-T
CC71
MOK006
MOK098
MOK128
CCL20 fold change in expression (log2)
Time (h)
CC97
MOK023
MOK028
MOK032
CCL20 fold change in gene expression (log2)
Time (h)
ST136
MOK076
MOK077
MOK083
CCL20 fold change in gene expression (log2)
Time (h)
CC151
MOK008
MOK043
MOK124
CCL20 fold change in gene expression (log2)
Time (h)

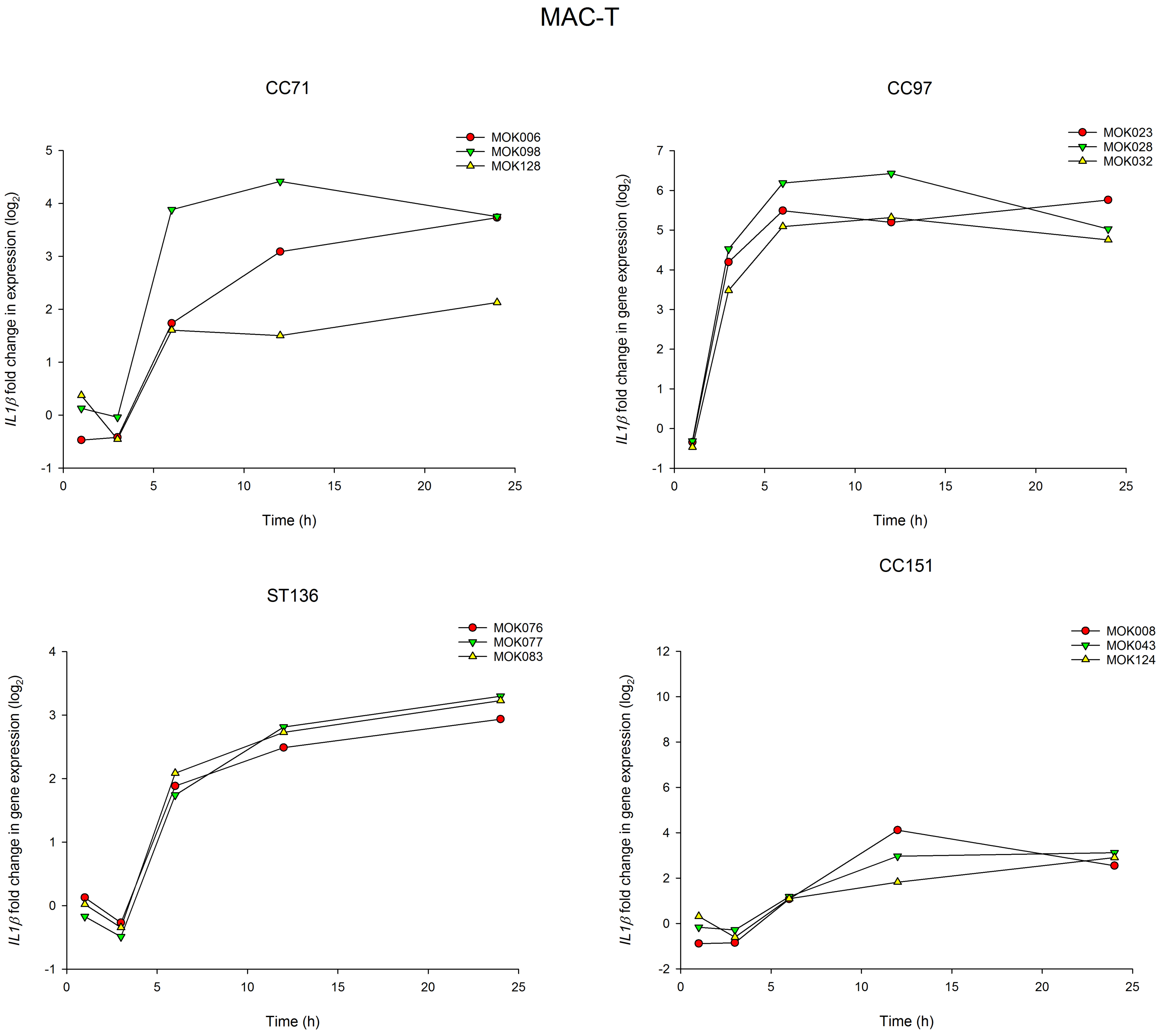
MAC-T
CC71
MOK006
MOK098
MOK128
IL1β fold change in expression (log2)
Time (h)
CC97
MOK023
MOK028
MOK032
IL1β fold change in gene expression (log2)
Time (h)
ST136
MOK076
MOK077
MOK083
IL1β fold change in gene expression (log2)
Time (h)
CC151
MOK008
MOK043
MOK124
IL1β fold change in gene expression (log2)
Time (h)

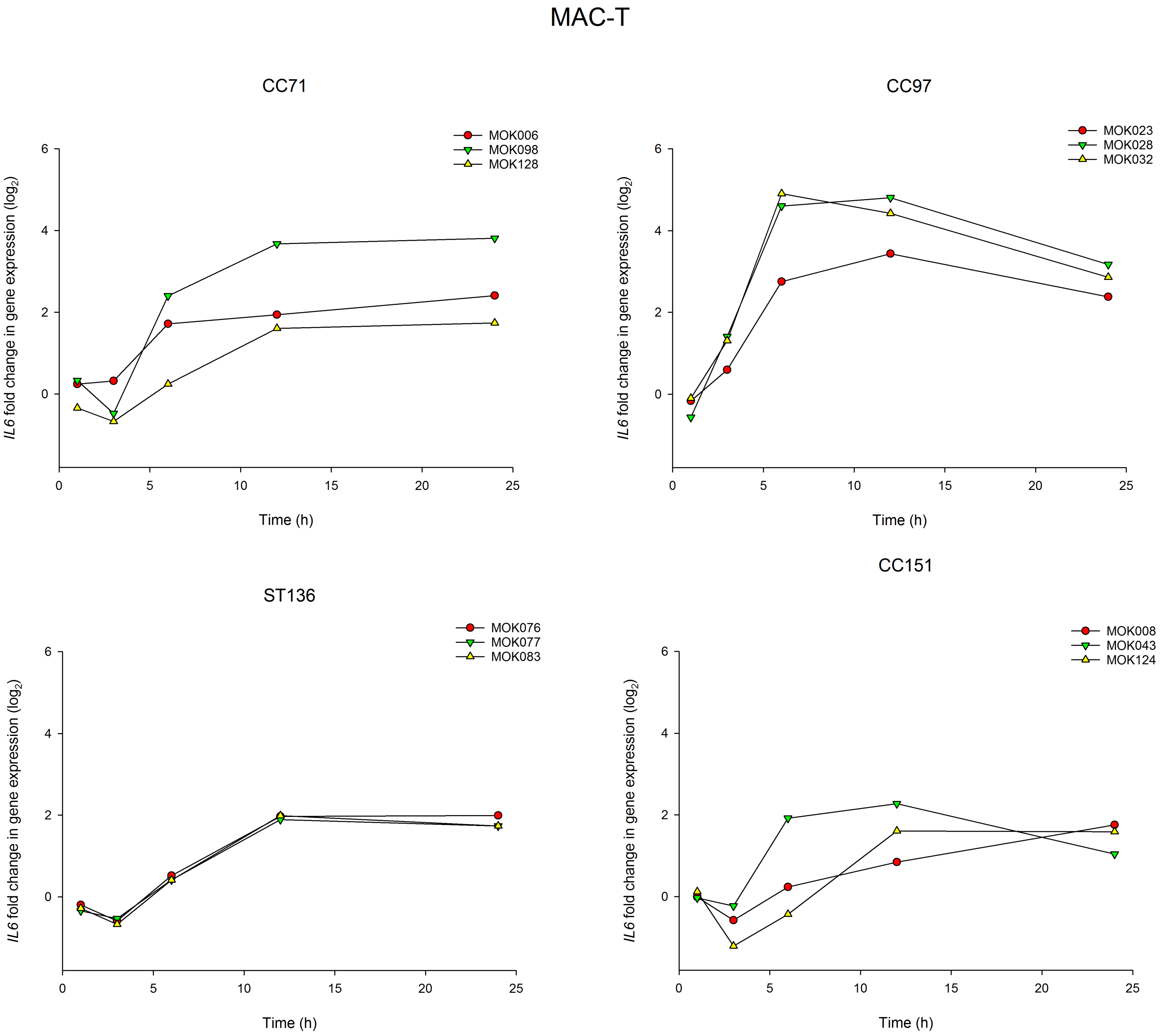
MAC-T
CC71
MOK006
MOK098
MOK128
CC97
MOK023
MOK028
MOK032
ST136
MOK076
MOK077
MOK083
CC151
MOK008
MOK043
MOK124
IL6 fold change in gene expression (log2)
Time (h)
0
2
4
6
5
10
15
20
25

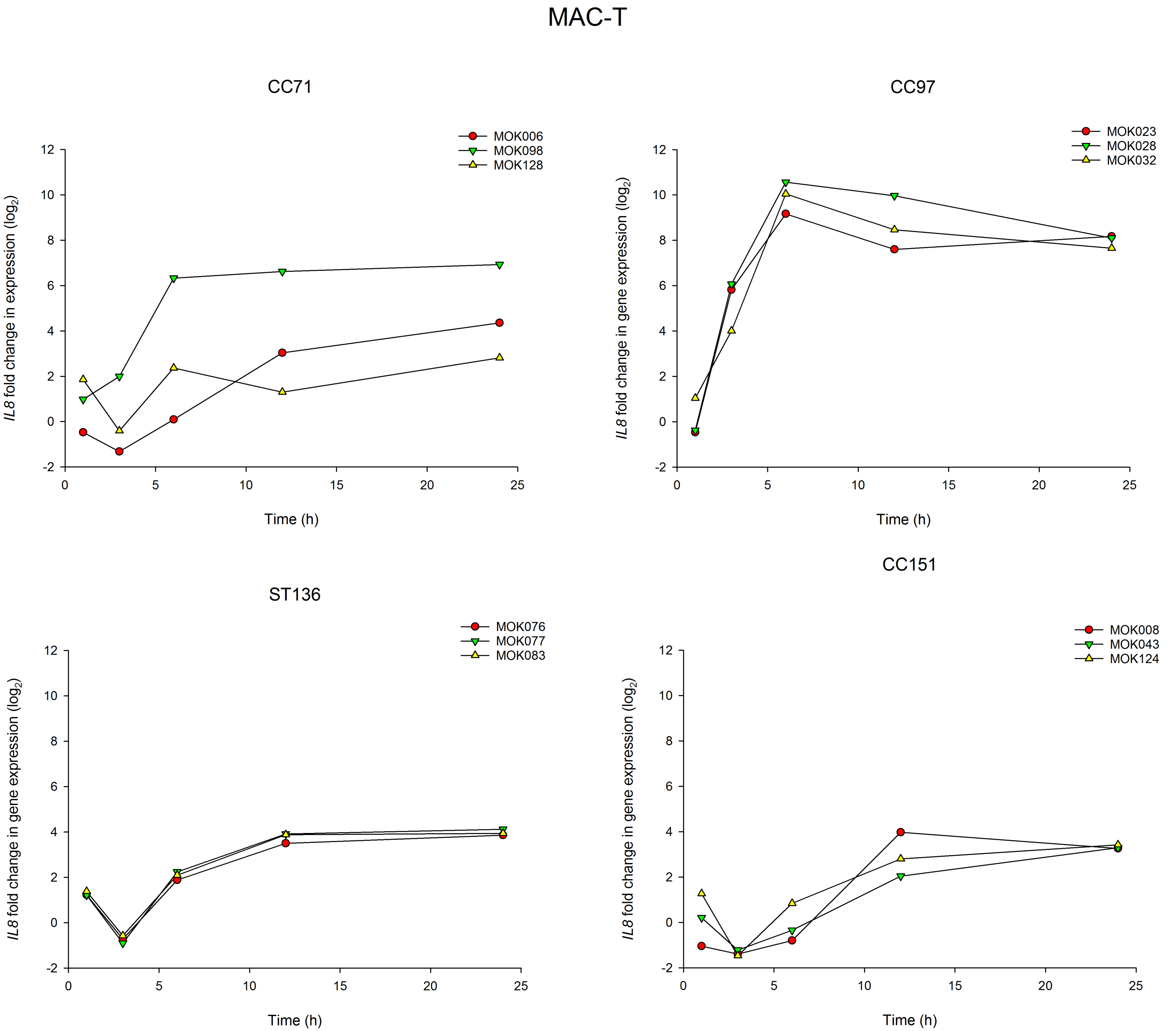
MAC-T
CC71
MOK006
MOK098
MOK128
IL8 fold change in expression (log2)
Time (h)
CC97
MOK023
MOK028
MOK032
IL8 fold change in gene expression (log2)
Time (h)
ST136
MOK076
MOK077
MOK083
IL8 fold change in gene expression (log2)
Time (h)
CC151
MOK008
MOK043
MOK124
IL8 fold change in gene expression (log2)
Time (h)

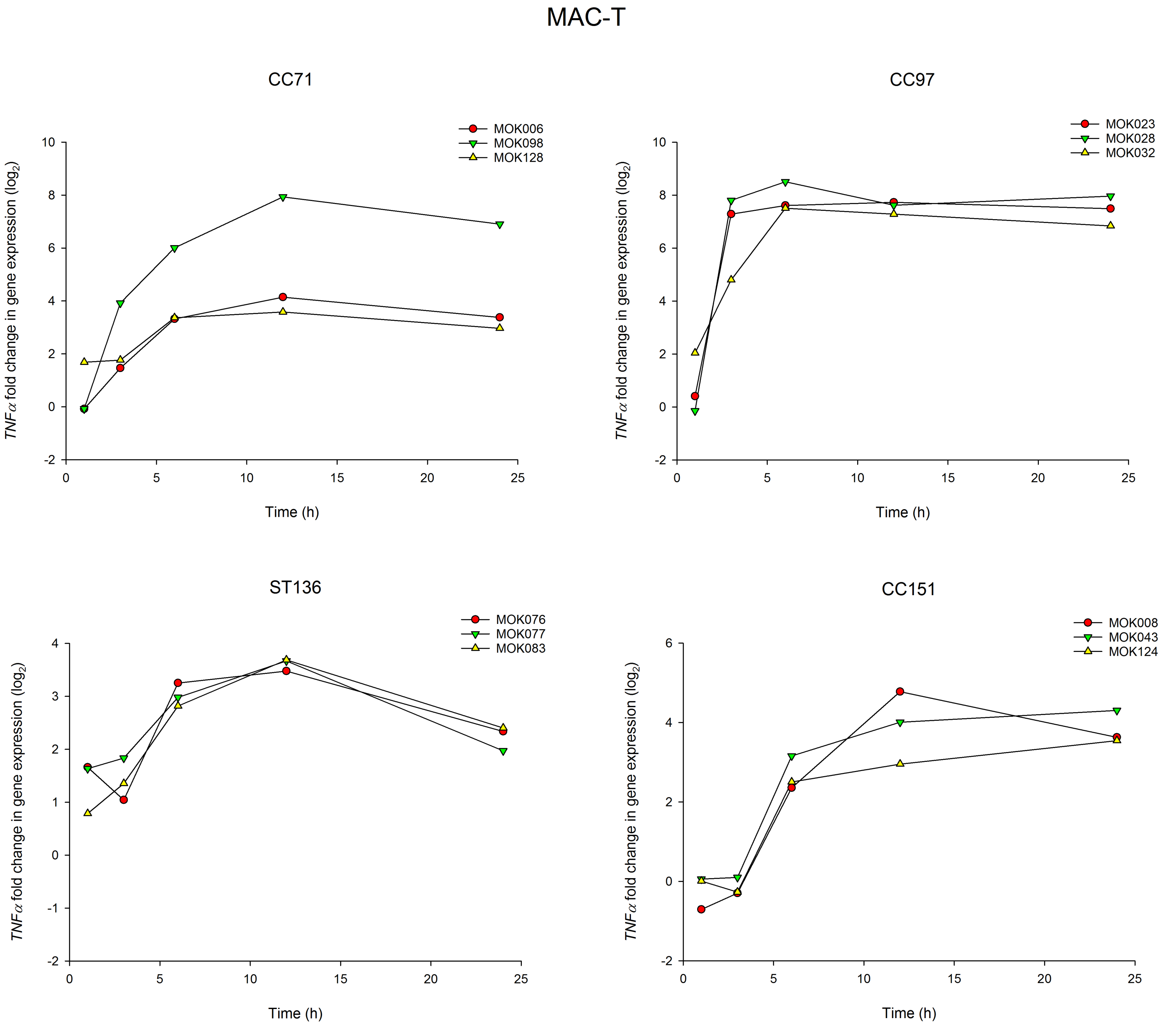
MAC-T
CC71
MOK006
MOK098
MOK128
CC97
MOK023
MOK028
MOK032
ST136
MOK076
MOK077
MOK083
CC151
MOK008
MOK043
MOK124
TNFα fold change in gene expression (log2)
Time (h)

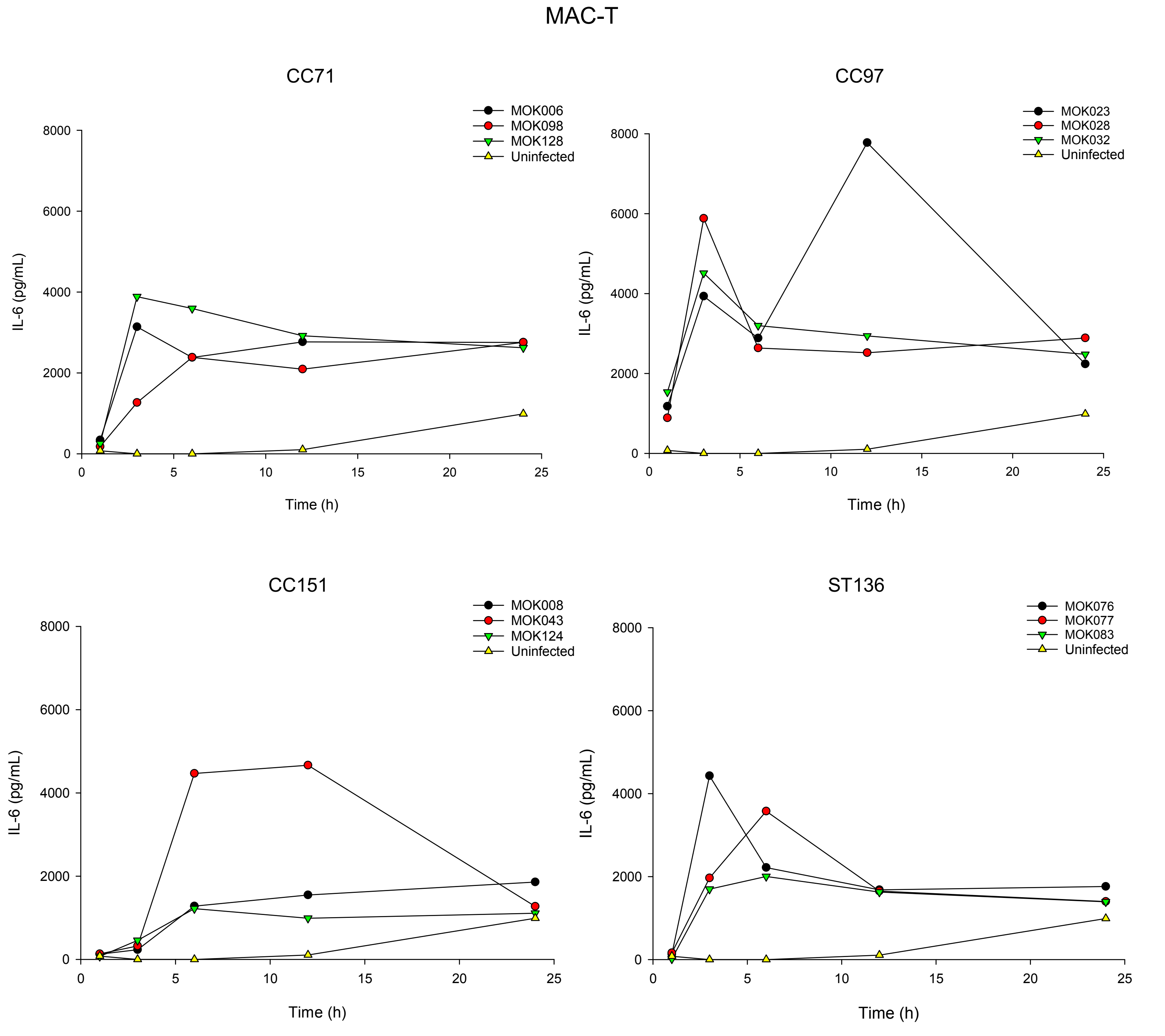
MAC-T
CC71
MOK006
MOK098
MOK128
Uninfected
IL-6 (pg/mL)
Time (h)
CC97
MOK023
MOK028
MOK032
Uninfected
IL-6 (pg/mL)
Time (h)
CC151
MOK008
MOK043
MOK124
Uninfected
IL-6 (pg/mL)
Time (h)
ST136
MOK076
MOK077
MOK083
Uninfected
IL-6 (pg/mL)
Time (h)

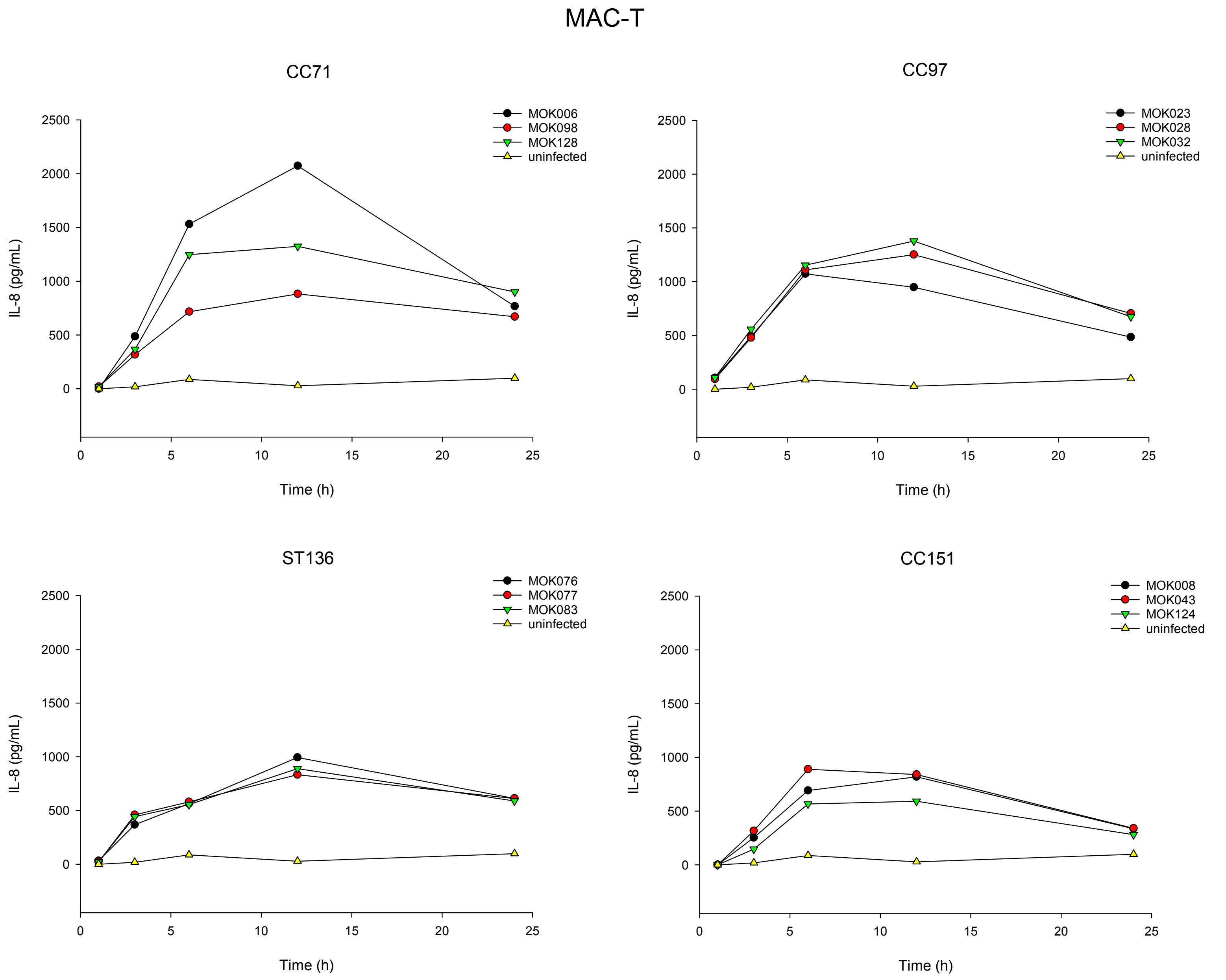
MAC-T
CC71
MOK006
MOK098
MOK128
uninfected
CC97
MOK023
MOK028
MOK032
uninfected
ST136
MOK076
MOK077
MOK083
uninfected
CC151
MOK008
MOK043
MOK124
uninfected
IL-8 (pg/mL)
Time (h)

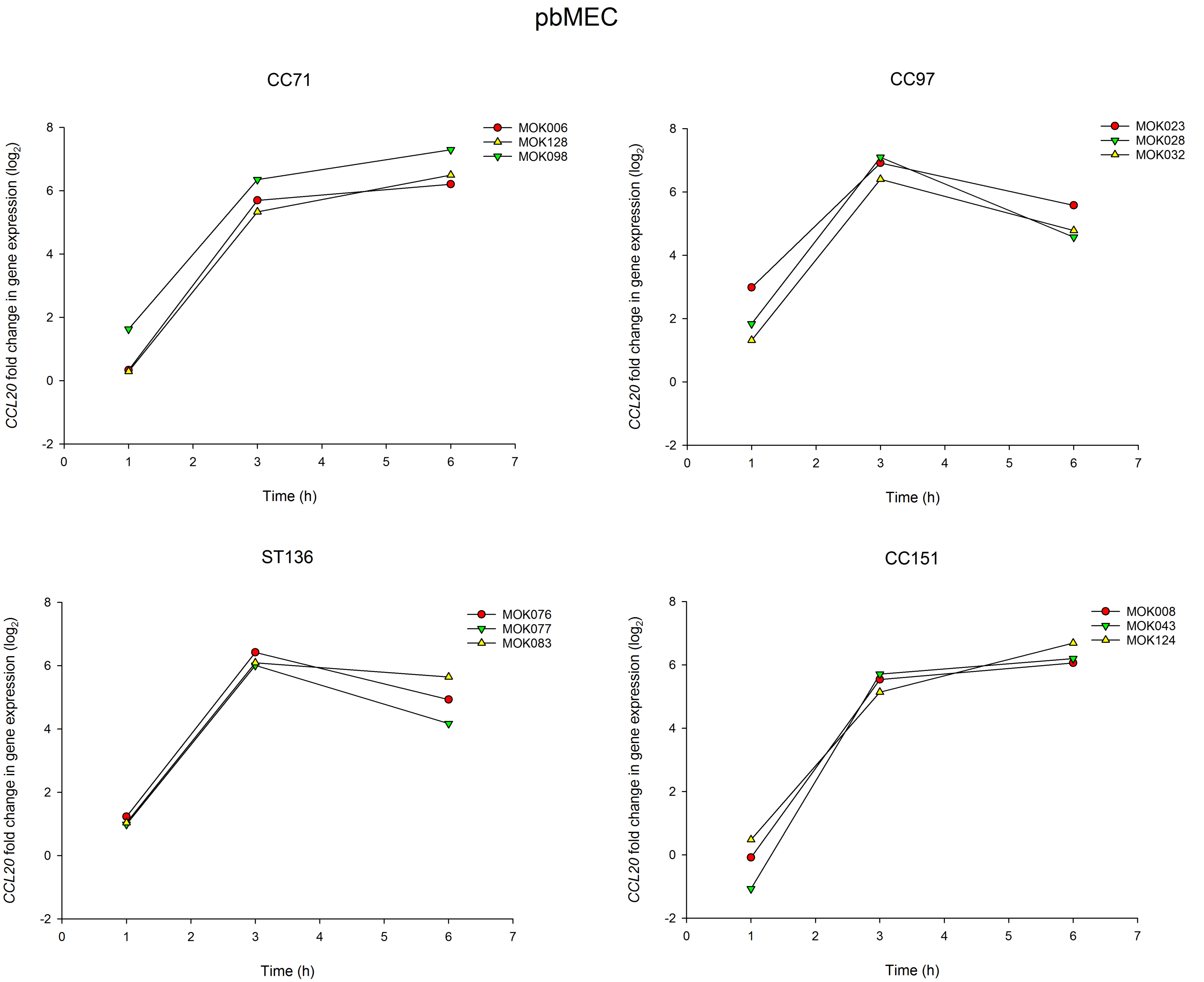
pbMEC
CC71
MOK006
MOK128
MOK098
CC97
MOK023
MOK028
MOK032
ST136
MOK076
MOK077
MOK083
CC151
MOK008
MOK043
MOK124
CCL20 fold change in gene expression (log2)
Time (h)
-2
0
2
4
6
8
0
1
2
3
4
5
6
7

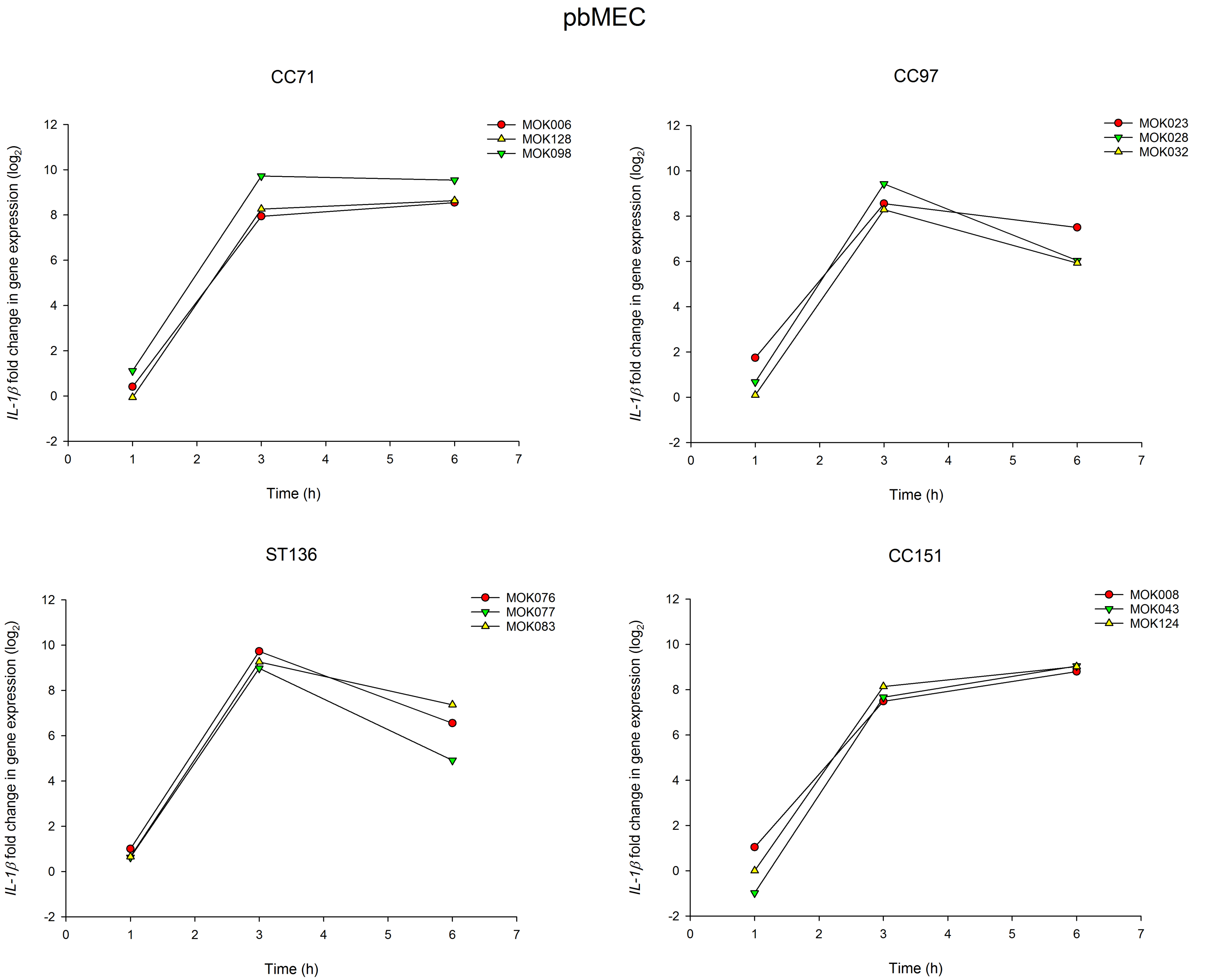

pbMEC
CC71
MOK006
MOK128
MOK098
CC97
MOK023
MOK028
MOK032
ST136
MOK076
MOK077
MOK083
CC151
MOK008
MOK043
MOK124
IL-1β fold change in gene expression (log2)
Time (h)

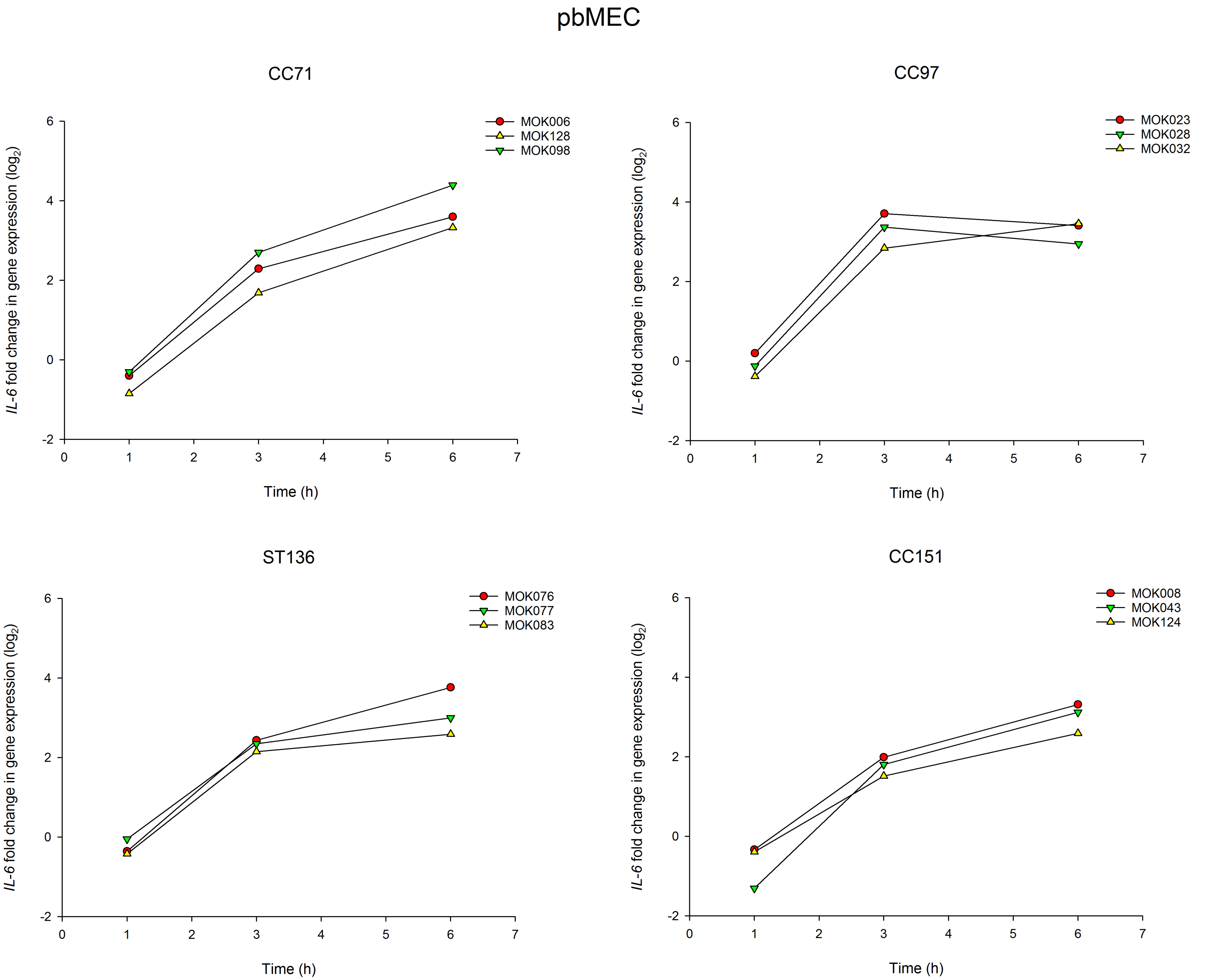
pbMEC
CC71
MOK006
MOK128
MOK098
CC97
MOK023
MOK028
MOK032
ST136
MOK076
MOK077
MOK083
CC151
MOK008
MOK043
MOK124
IL-6 fold change in gene expression (log2)
Time (h)

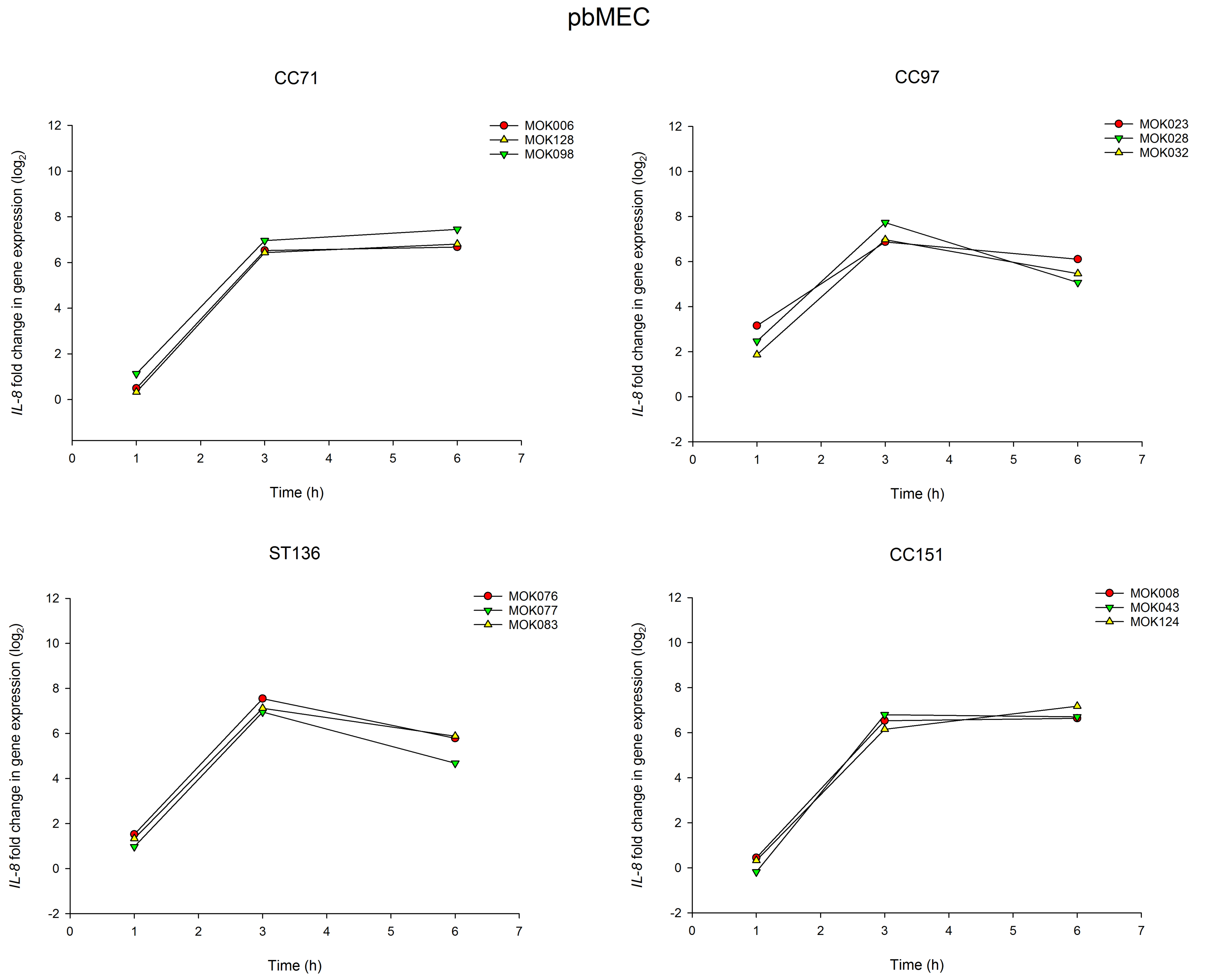

pbMEC
CC71
MOK006
MOK128
MOK098
IL-8 fold change in gene expression (log2)
Time (h)
CC97
MOK023
MOK028
MOK032
ST136
MOK076
MOK077
MOK083
CC151
MOK008
MOK043
MOK124

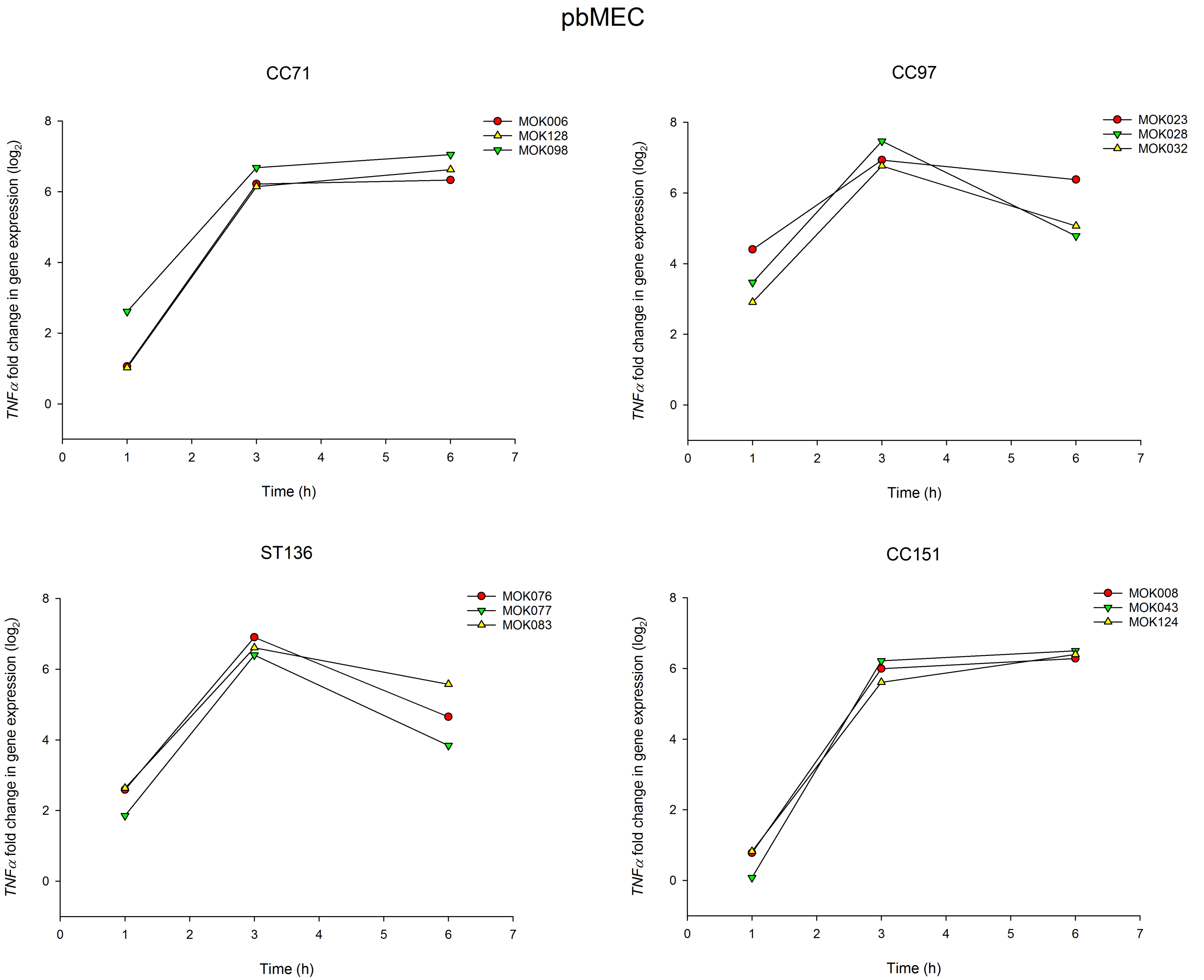

pbMEC
CC71
MOK006
MOK128
MOK098
CC97
MOK023
MOK028
MOK032
ST136
MOK076
MOK077
MOK083
CC151
MOK008
MOK043
MOK124
TNFα fold change in gene expression (log2)
Time (h)

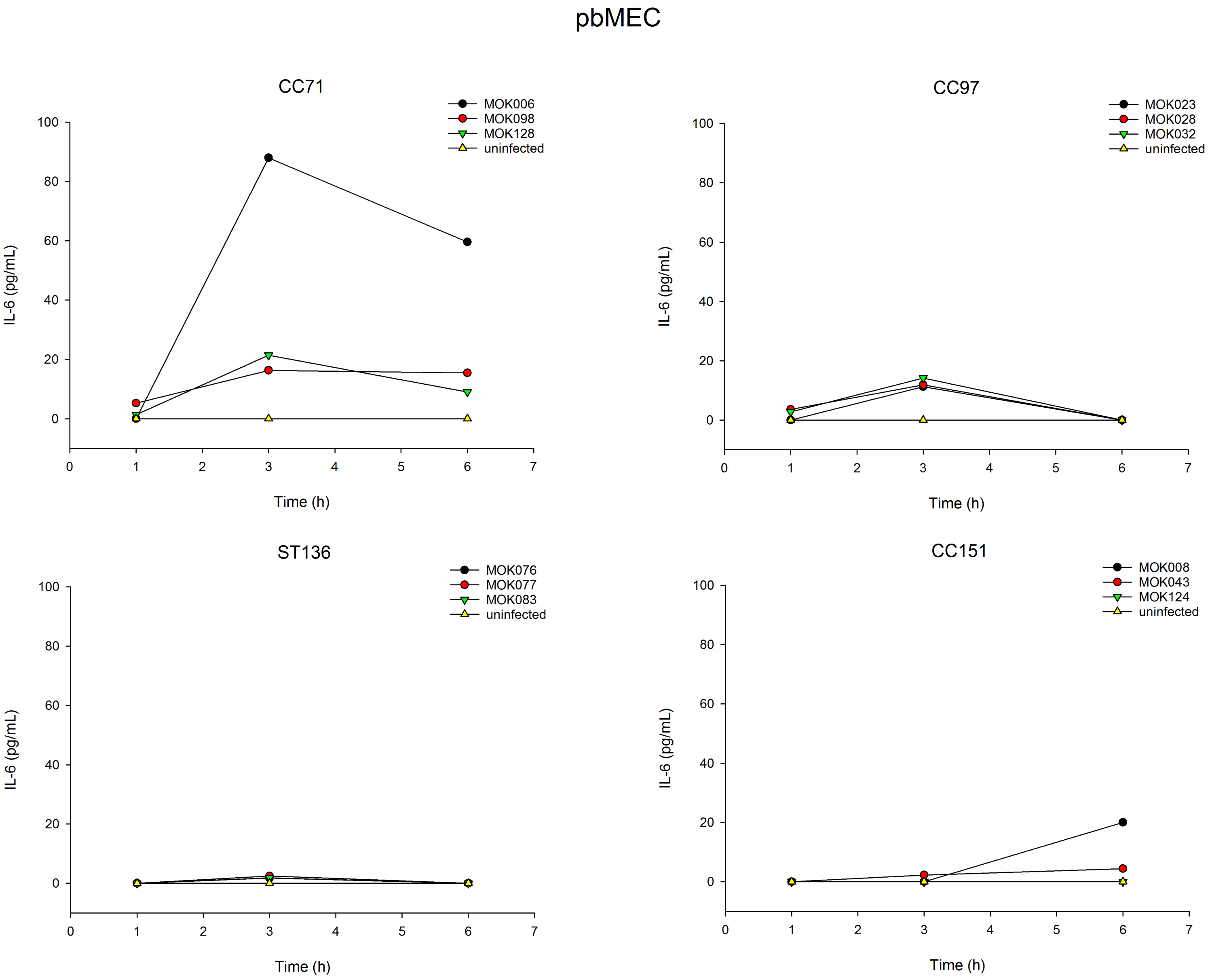
pbMEC
CC71
MOK006
MOK098
MOK128
uninfected
IL-6 (pg/mL)
Time (h)
CC97
MOK023
MOK028
MOK032
uninfected
IL-6 (pg/mL)
Time (h)
ST136
MOK076
MOK077
MOK083
uninfected
IL-6 (pg/mL)
Time (h)
CC151
MOK008
MOK043
MOK124
uninfected
IL-6 (pg/mL)
Time (h)

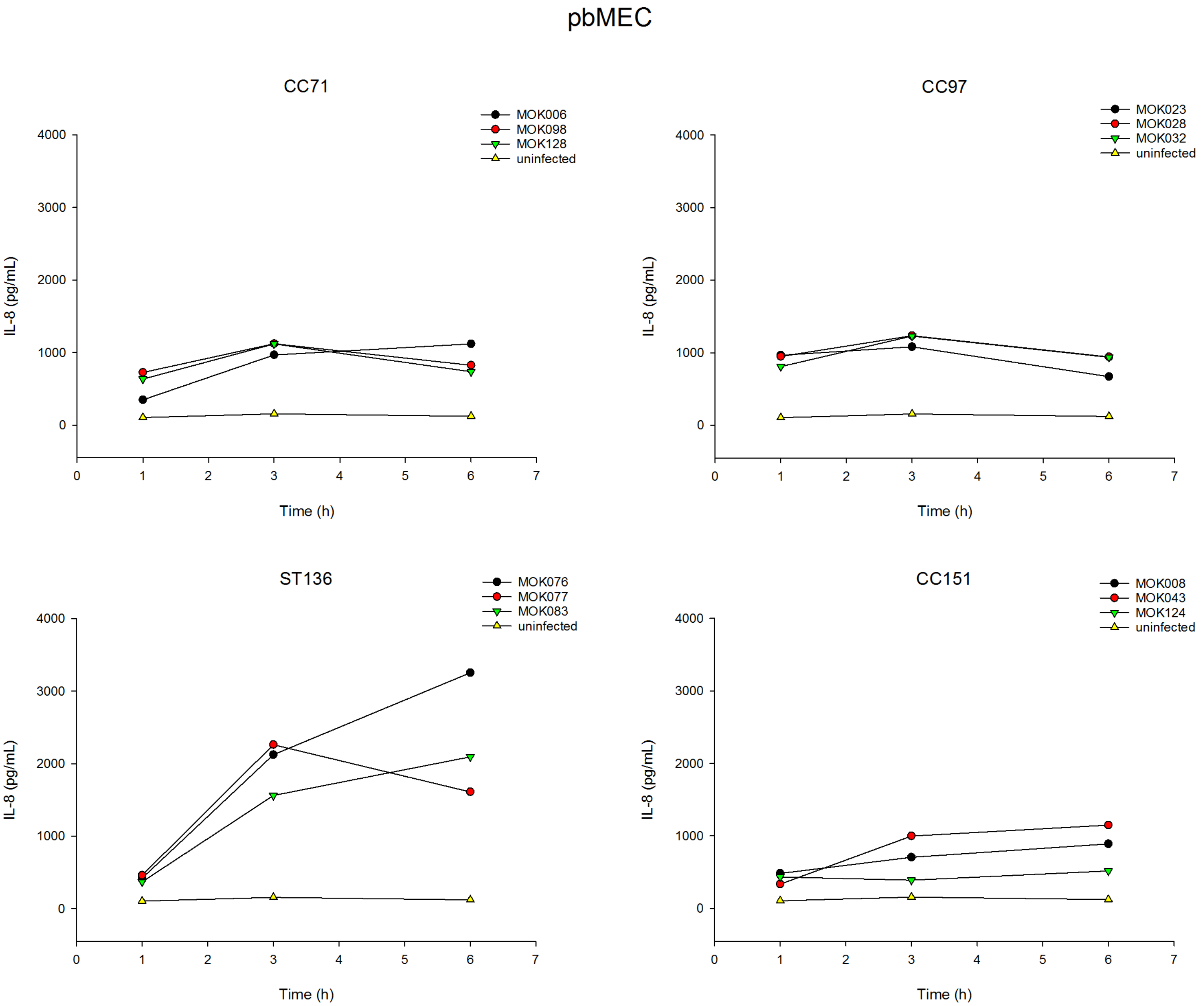

pbMEC
CC71
MOK006
MOK098
MOK128
uninfected
CC97
MOK023
MOK028
MOK032
uninfected
ST136
MOK076
MOK077
MOK083
uninfected
CC151
MOK008
MOK043
MOK124
uninfected
IL-8 (pg/mL)
Time (h)

Significant differences in *CCL20* gene expression in MAC-T infected with *S. aureus*

Time	Strain	Strain	Gene	Adj P
3	MOK006	MOK023	*CCL20*	<.0001
3	MOK006	MOK028	*CCL20*	<.0001
3	MOK006	MOK032	*CCL20*	<.0001
3	MOK006	MOK098	*CCL20*	0.0205
3	MOK008	MOK023	*CCL20*	<.0001
3	MOK008	MOK028	*CCL20*	<.0001
3	MOK008	MOK032	*CCL20*	<.0001
3	MOK008	MOK076	*CCL20*	0.0488
3	MOK008	MOK077	*CCL20*	0.0485
3	MOK008	MOK098	*CCL20*	<.0001
3	MOK008	MOK128	*CCL20*	0.0009
3	MOK023	MOK043	*CCL20*	<.0001
3	MOK023	MOK076	*CCL20*	<.0001
3	MOK023	MOK077	*CCL20*	<.0001
3	MOK023	MOK083	*CCL20*	<.0001
3	MOK023	MOK098	*CCL20*	0.0042
3	MOK023	MOK124	*CCL20*	<.0001
3	MOK023	MOK128	*CCL20*	<.0001
3	MOK028	MOK043	*CCL20*	<.0001
3	MOK028	MOK076	*CCL20*	<.0001
3	MOK028	MOK077	*CCL20*	<.0001
3	MOK028	MOK083	*CCL20*	<.0001
3	MOK028	MOK098	*CCL20*	0.0018
3	MOK028	MOK124	*CCL20*	<.0001
3	MOK028	MOK128	*CCL20*	<.0001
3	MOK032	MOK043	*CCL20*	<.0001
3	MOK032	MOK076	*CCL20*	<.0001
3	MOK032	MOK077	*CCL20*	<.0001
3	MOK032	MOK083	*CCL20*	<.0001
3	MOK032	MOK098	*CCL20*	0.0288
3	MOK032	MOK124	*CCL20*	<.0001
3	MOK032	MOK128	*CCL20*	<.0001
3	MOK043	MOK098	*CCL20*	<.0001
3	MOK076	MOK098	*CCL20*	0.0235
3	MOK077	MOK098	*CCL20*	0.0237
3	MOK083	MOK098	*CCL20*	0.0156
3	MOK098	MOK124	*CCL20*	<.0001
3	MOK124	MOK128	*CCL20*	0.0025
6	MOK006	MOK023	*CCL20*	<.0001
6	MOK006	MOK028	*CCL20*	<.0001
6	MOK006	MOK032	*CCL20*	<.0001
6	MOK006	MOK098	*CCL20*	<.0001
6	MOK008	MOK023	*CCL20*	<.0001
6	MOK008	MOK028	*CCL20*	<.0001
6	MOK008	MOK032	*CCL20*	<.0001
6	MOK008	MOK077	*CCL20*	0.0195
6	MOK008	MOK098	*CCL20*	<.0001
6	MOK023	MOK043	*CCL20*	<.0001

6	MOK023	MOK076	*CCL20*	<.0001
6	MOK023	MOK077	*CCL20*	<.0001
6	MOK023	MOK083	*CCL20*	<.0001
6	MOK023	MOK124	*CCL20*	<.0001
6	MOK023	MOK128	*CCL20*	<.0001
6	MOK028	MOK043	*CCL20*	<.0001
6	MOK028	MOK076	*CCL20*	<.0001
6	MOK028	MOK077	*CCL20*	<.0001
6	MOK028	MOK083	*CCL20*	<.0001
6	MOK028	MOK124	*CCL20*	<.0001
6	MOK028	MOK128	*CCL20*	<.0001
6	MOK032	MOK043	*CCL20*	<.0001
6	MOK032	MOK076	*CCL20*	<.0001
6	MOK032	MOK077	*CCL20*	<.0001
6	MOK032	MOK083	*CCL20*	<.0001
6	MOK032	MOK124	*CCL20*	<.0001
6	MOK032	MOK128	*CCL20*	<.0001
6	MOK043	MOK098	*CCL20*	<.0001
6	MOK076	MOK098	*CCL20*	0.0002
6	MOK077	MOK098	*CCL20*	0.0009
6	MOK077	MOK124	*CCL20*	0.0275
6	MOK083	MOK098	*CCL20*	<.0001
6	MOK098	MOK124	*CCL20*	<.0001
6	MOK098	MOK128	*CCL20*	<.0001
12	MOK006	MOK023	*CCL20*	0.0387
12	MOK006	MOK028	*CCL20*	0.0013
12	MOK006	MOK032	*CCL20*	0.0205
12	MOK006	MOK098	*CCL20*	0.0053
12	MOK023	MOK076	*CCL20*	0.0332
12	MOK023	MOK077	*CCL20*	0.0157
12	MOK023	MOK083	*CCL20*	0.0212
12	MOK023	MOK128	*CCL20*	0.0007
12	MOK028	MOK043	*CCL20*	0.0018
12	MOK028	MOK076	*CCL20*	0.0011
12	MOK028	MOK077	*CCL20*	0.0004
12	MOK028	MOK083	*CCL20*	0.0006
12	MOK028	MOK124	*CCL20*	0.0027
12	MOK028	MOK128	*CCL20*	<.0001
12	MOK032	MOK043	*CCL20*	0.028
12	MOK032	MOK076	*CCL20*	0.0174
12	MOK032	MOK077	*CCL20*	0.008
12	MOK032	MOK083	*CCL20*	0.0109
12	MOK032	MOK124	*CCL20*	0.0396
12	MOK032	MOK128	*CCL20*	0.0003
12	MOK043	MOK098	*CCL20*	0.0075
12	MOK076	MOK098	*CCL20*	0.0045
12	MOK077	MOK098	*CCL20*	0.002
12	MOK083	MOK098	*CCL20*	0.0027
12	MOK098	MOK124	*CCL20*	0.0109
12	MOK098	MOK128	*CCL20*	<.0001

24	MOK006	MOK043	*CCL20*	0.0118
24	MOK006	MOK076	*CCL20*	0.011
24	MOK006	MOK077	*CCL20*	0.0001
24	MOK006	MOK083	*CCL20*	0.002
24	MOK006	MOK124	*CCL20*	0.0321
24	MOK006	MOK128	*CCL20*	0.0005
24	MOK008	MOK023	*CCL20*	<.0001
24	MOK008	MOK028	*CCL20*	0.0003
24	MOK008	MOK098	*CCL20*	<.0001
24	MOK023	MOK043	*CCL20*	<.0001
24	MOK023	MOK076	*CCL20*	<.0001
24	MOK023	MOK077	*CCL20*	<.0001
24	MOK023	MOK083	*CCL20*	<.0001
24	MOK023	MOK124	*CCL20*	<.0001
24	MOK023	MOK128	*CCL20*	<.0001
24	MOK028	MOK043	*CCL20*	<.0001
24	MOK028	MOK076	*CCL20*	<.0001
24	MOK028	MOK077	*CCL20*	<.0001
24	MOK028	MOK083	*CCL20*	<.0001
24	MOK028	MOK124	*CCL20*	0.0002
24	MOK028	MOK128	*CCL20*	<.0001
24	MOK032	MOK043	*CCL20*	0.0202
24	MOK032	MOK076	*CCL20*	0.0188
24	MOK032	MOK077	*CCL20*	0.0002
24	MOK032	MOK083	*CCL20*	0.0037
24	MOK032	MOK128	*CCL20*	0.0009
24	MOK043	MOK098	*CCL20*	<.0001
24	MOK076	MOK098	*CCL20*	<.0001
24	MOK077	MOK098	*CCL20*	<.0001
24	MOK083	MOK098	*CCL20*	<.0001
24	MOK098	MOK124	*CCL20*	<.0001
24	MOK098	MOK128	*CCL20*	<.0001

Significant differences in *IL1 β* gene expression in MAC-T infected with *S. aureus*

Time	Strain	Strain	Gene	Adj P
3	MOK006	MOK023	*IL1B*	0.0004
3	MOK006	MOK028	*IL1B*	0.0001
3	MOK006	MOK032	*IL1B*	0.0039
3	MOK008	MOK023	*IL1B*	<.0001
3	MOK008	MOK028	*IL1B*	<.0001
3	MOK008	MOK032	*IL1B*	0.0009
3	MOK023	MOK043	*IL1B*	0.0006
3	MOK023	MOK076	*IL1B*	0.0006
3	MOK023	MOK077	*IL1B*	0.0003
3	MOK023	MOK083	*IL1B*	0.0021
3	MOK023	MOK098	*IL1B*	0.0013
3	MOK023	MOK124	*IL1B*	0.0002
3	MOK023	MOK128	*IL1B*	0.0003
3	MOK028	MOK043	*IL1B*	0.0002
3	MOK028	MOK076	*IL1B*	0.0002
3	MOK028	MOK077	*IL1B*	0.0001
3	MOK028	MOK083	*IL1B*	0.0008
3	MOK028	MOK098	*IL1B*	0.0005
3	MOK028	MOK124	*IL1B*	<.0001
3	MOK028	MOK128	*IL1B*	0.0001
3	MOK032	MOK043	*IL1B*	0.0058
3	MOK032	MOK076	*IL1B*	0.0062
3	MOK032	MOK077	*IL1B*	0.0031
3	MOK032	MOK083	*IL1B*	0.0155
3	MOK032	MOK098	*IL1B*	0.0126
3	MOK032	MOK124	*IL1B*	0.0021
3	MOK032	MOK128	*IL1B*	0.0035
6	MOK006	MOK023	*IL1B*	<.0001
6	MOK006	MOK028	*IL1B*	<.0001
6	MOK006	MOK032	*IL1B*	0.0003
6	MOK008	MOK023	*IL1B*	<.0001
6	MOK008	MOK028	*IL1B*	<.0001
6	MOK008	MOK032	*IL1B*	<.0001
6	MOK008	MOK098	*IL1B*	0.0035
6	MOK023	MOK043	*IL1B*	<.0001
6	MOK023	MOK076	*IL1B*	<.0001
6	MOK023	MOK077	*IL1B*	0.0004
6	MOK023	MOK083	*IL1B*	0.0002
6	MOK023	MOK124	*IL1B*	<.0001
6	MOK023	MOK128	*IL1B*	0.0002
6	MOK028	MOK043	*IL1B*	<.0001
6	MOK028	MOK076	*IL1B*	<.0001
6	MOK028	MOK077	*IL1B*	<.0001
6	MOK028	MOK083	*IL1B*	<.0001
6	MOK028	MOK098	*IL1B*	0.0308
6	MOK028	MOK124	*IL1B*	<.0001
6	MOK028	MOK128	*IL1B*	<.0001
6	MOK032	MOK043	*IL1B*	<.0001

6	MOK032	MOK076	*IL1B*	0.0006
6	MOK032	MOK077	*IL1B*	0.0022
6	MOK032	MOK083	*IL1B*	0.0015
6	MOK032	MOK124	*IL1B*	<.0001
6	MOK032	MOK128	*IL1B*	0.0012
6	MOK043	MOK098	*IL1B*	0.0056
6	MOK098	MOK124	*IL1B*	0.0038

Significant differences in *IL6* gene expression in MAC-T cells with *S. aureus*

**Time**	**Strain**	**Strain**	**Gene**	**Adj P**
6	MOK028	MOK124	*IL6*	0.0416
6	MOK032	MOK124	*IL6*	0.0241
12	MOK008	MOK028	*IL6*	0.0014
12	MOK008	MOK032	*IL6*	0.0052
12	MOK028	MOK077	*IL6*	0.0447
12	MOK028	MOK124	*IL6*	0.0184
12	MOK028	MOK128	*IL6*	0.0183
24	MOK043	MOK098	*IL6*	0.047

Significant differences in *IL8* gene expression in MAC-T infected with *S. aureus*

Time	Strain	Strain	Gene	Adj P
3	MOK006	MOK023	*IL8*	<.0001
3	MOK006	MOK028	*IL8*	<.0001
3	MOK006	MOK032	*IL8*	0.0046
3	MOK008	MOK023	*IL8*	<.0001
3	MOK008	MOK028	*IL8*	<.0001
3	MOK008	MOK032	*IL8*	0.0038
3	MOK023	MOK043	*IL8*	<.0001
3	MOK023	MOK076	*IL8*	0.0002
3	MOK023	MOK077	*IL8*	0.0002
3	MOK023	MOK083	*IL8*	0.0003
3	MOK023	MOK124	*IL8*	<.0001
3	MOK023	MOK128	*IL8*	0.0005
3	MOK028	MOK043	*IL8*	<.0001
3	MOK028	MOK076	*IL8*	0.0001
3	MOK028	MOK077	*IL8*	<.0001
3	MOK028	MOK083	*IL8*	0.0002
3	MOK028	MOK124	*IL8*	<.0001
3	MOK028	MOK128	*IL8*	0.0003
3	MOK032	MOK043	*IL8*	0.0057
3	MOK032	MOK076	*IL8*	0.0178
3	MOK032	MOK077	*IL8*	0.0119
3	MOK032	MOK083	*IL8*	0.0242
3	MOK032	MOK124	*IL8*	0.0032
3	MOK032	MOK128	*IL8*	0.0348
6	MOK006	MOK023	*IL8*	<.0001
6	MOK006	MOK028	*IL8*	<.0001
6	MOK006	MOK032	*IL8*	<.0001
6	MOK006	MOK098	*IL8*	0.0001
6	MOK008	MOK023	*IL8*	<.0001
6	MOK008	MOK028	*IL8*	<.0001
6	MOK008	MOK032	*IL8*	<.0001
6	MOK008	MOK098	*IL8*	<.0001
6	MOK023	MOK043	*IL8*	<.0001
6	MOK023	MOK076	*IL8*	<.0001
6	MOK023	MOK077	*IL8*	<.0001
6	MOK023	MOK083	*IL8*	<.0001
6	MOK023	MOK124	*IL8*	<.0001
6	MOK023	MOK128	*IL8*	<.0001
6	MOK028	MOK043	*IL8*	<.0001
6	MOK028	MOK076	*IL8*	<.0001
6	MOK028	MOK077	*IL8*	<.0001
6	MOK028	MOK083	*IL8*	<.0001
6	MOK028	MOK098	*IL8*	0.0222
6	MOK028	MOK124	*IL8*	<.0001
6	MOK028	MOK128	*IL8*	<.0001
6	MOK032	MOK043	*IL8*	<.0001
6	MOK032	MOK076	*IL8*	<.0001
6	MOK032	MOK077	*IL8*	<.0001

6	MOK032	MOK083	*IL8*	<.0001
6	MOK032	MOK124	*IL8*	<.0001
6	MOK032	MOK128	*IL8*	<.0001
6	MOK043	MOK098	*IL8*	<.0001
6	MOK076	MOK098	*IL8*	0.0136
6	MOK077	MOK098	*IL8*	0.0317
6	MOK083	MOK098	*IL8*	0.0227
6	MOK098	MOK124	*IL8*	0.0009
6	MOK098	MOK128	*IL8*	0.043
12	MOK006	MOK023	*IL8*	0.0033
12	MOK006	MOK028	*IL8*	<.0001
12	MOK006	MOK032	*IL8*	0.0003
12	MOK006	MOK098	*IL8*	0.0442
12	MOK008	MOK023	*IL8*	0.0401
12	MOK008	MOK028	*IL8*	<.0001
12	MOK008	MOK032	*IL8*	0.0041
12	MOK023	MOK043	*IL8*	0.0002
12	MOK023	MOK076	*IL8*	0.0118
12	MOK023	MOK077	*IL8*	0.0342
12	MOK023	MOK083	*IL8*	0.0318
12	MOK023	MOK124	*IL8*	0.0017
12	MOK023	MOK128	*IL8*	<.0001
12	MOK028	MOK043	*IL8*	<.0001
12	MOK028	MOK076	*IL8*	<.0001
12	MOK028	MOK077	*IL8*	<.0001
12	MOK028	MOK083	*IL8*	<.0001
12	MOK028	MOK124	*IL8*	<.0001
12	MOK028	MOK128	*IL8*	<.0001
12	MOK032	MOK043	*IL8*	<.0001
12	MOK032	MOK076	*IL8*	0.001
12	MOK032	MOK077	*IL8*	0.0034
12	MOK032	MOK083	*IL8*	0.0031
12	MOK032	MOK124	*IL8*	0.0001
12	MOK032	MOK128	*IL8*	<.0001
12	MOK043	MOK098	*IL8*	0.0031
12	MOK098	MOK124	*IL8*	0.0243
12	MOK098	MOK128	*IL8*	0.0004
24	MOK006	MOK023	*IL8*	0.0418
24	MOK008	MOK023	*IL8*	0.0026
24	MOK008	MOK028	*IL8*	0.0032
24	MOK008	MOK032	*IL8*	0.0103
24	MOK023	MOK043	*IL8*	0.0028
24	MOK023	MOK076	*IL8*	0.0124
24	MOK023	MOK077	*IL8*	0.0237
24	MOK023	MOK083	*IL8*	0.0153
24	MOK023	MOK124	*IL8*	0.004
24	MOK023	MOK128	*IL8*	0.0008
24	MOK028	MOK043	*IL8*	0.0035
24	MOK028	MOK076	*IL8*	0.0151
24	MOK028	MOK077	*IL8*	0.0286

24	MOK028	MOK083	*IL8*	0.0185
24	MOK028	MOK124	*IL8*	0.0049
24	MOK028	MOK128	*IL8*	0.001
24	MOK032	MOK043	*IL8*	0.0111
24	MOK032	MOK076	*IL8*	0.0438
24	MOK032	MOK124	*IL8*	0.0153
24	MOK032	MOK128	*IL8*	0.0032
24	MOK098	MOK128	*IL8*	0.0211

Significant differences in *TNF α* gene expression in MAC-T infected with *S. aureus*

Time	Strain	Strain	Gene	Adj P
3	MOK006	MOK023	*TNF*	0.0276
3	MOK006	MOK028	*TNF*	0.0114
3	MOK008	MOK023	*TNF*	0.0012
3	MOK008	MOK028	*TNF*	0.0004
3	MOK023	MOK043	*TNF*	0.0025
3	MOK023	MOK076	*TNF*	0.0136
3	MOK023	MOK083	*TNF*	0.023
3	MOK023	MOK124	*TNF*	0.0012
3	MOK023	MOK128	*TNF*	0.045
3	MOK028	MOK043	*TNF*	0.0009
3	MOK028	MOK076	*TNF*	0.0054
3	MOK028	MOK077	*TNF*	0.0216
3	MOK028	MOK083	*TNF*	0.0094
3	MOK028	MOK124	*TNF*	0.0005
3	MOK028	MOK128	*TNF*	0.0192
6	MOK006	MOK023	*TNF*	0.031
6	MOK006	MOK028	*TNF*	0.0038
6	MOK006	MOK032	*TNF*	0.0386
6	MOK008	MOK023	*TNF*	0.0032
6	MOK008	MOK028	*TNF*	0.0003
6	MOK008	MOK032	*TNF*	0.0041
6	MOK023	MOK043	*TNF*	0.0216
6	MOK023	MOK076	*TNF*	0.0265
6	MOK023	MOK077	*TNF*	0.0144
6	MOK023	MOK083	*TNF*	0.0098
6	MOK023	MOK124	*TNF*	0.0046
6	MOK023	MOK128	*TNF*	0.0343
6	MOK028	MOK043	*TNF*	0.0025
6	MOK028	MOK076	*TNF*	0.0032
6	MOK028	MOK077	*TNF*	0.0016
6	MOK028	MOK083	*TNF*	0.0011
6	MOK028	MOK124	*TNF*	0.0005
6	MOK028	MOK128	*TNF*	0.0043
6	MOK032	MOK043	*TNF*	0.027
6	MOK032	MOK076	*TNF*	0.0331
6	MOK032	MOK077	*TNF*	0.0181
6	MOK032	MOK083	*TNF*	0.0125
6	MOK032	MOK124	*TNF*	0.0059
6	MOK032	MOK128	*TNF*	0.0426
24	MOK023	MOK076	*TNF*	0.041
24	MOK023	MOK077	*TNF*	0.0215
24	MOK023	MOK083	*TNF*	0.046
24	MOK028	MOK076	*TNF*	0.0177
24	MOK028	MOK077	*TNF*	0.0089
24	MOK028	MOK083	*TNF*	0.02

Significant differences in IL-6 protein expression in MAC-T infected with *S. aureus*

Time	Strain	Strain	Protein	Adj P
1	MOK023	MOK083	IL-6	0.0218
1	MOK023	MOK124	IL-6	0.0315
1	MOK028	MOK083	IL-6	0.031
1	MOK028	MOK124	IL-6	0.0447
1	MOK032	MOK076	IL-6	0.0178
1	MOK032	MOK077	IL-6	0.0306
1	MOK032	MOK083	IL-6	0.0059
1	MOK032	MOK098	IL-6	0.048
1	MOK032	MOK124	IL-6	0.0084
1	MOK032	MOK128	IL-6	0.0435
3	MOK008	MOK028	IL-6	0.0457
6	MOK008	MOK043	IL-6	0.0094
6	MOK043	MOK124	IL-6	0.0087
12	MOK006	MOK023	IL-6	0.0213
12	MOK008	MOK023	IL-6	0.0012
12	MOK023	MOK028	IL-6	0.013
12	MOK023	MOK032	IL-6	0.03
12	MOK023	MOK076	IL-6	0.0017
12	MOK023	MOK077	IL-6	0.0017
12	MOK023	MOK083	IL-6	0.0015
12	MOK023	MOK098	IL-6	0.0051
12	MOK023	MOK124	IL-6	0.0003
12	MOK023	MOK128	IL-6	0.0257
12	MOK043	MOK124	IL-6	0.0207

Significant differences in IL-8 protein expression in MAC-T infected with *S. aureus*

Time	Strain	Strain	Protein	Adj P
12	MOK006	MOK124	IL-8	0.0401

Significant differences in *TNFα* gene expression in primary bMEC infected with *S. aureus*

Time	Strain	Strain	Gene	Adj P
1	MOK006	MOK023	*TNFα*	0.0469
1	MOK008	MOK023	*TNFα*	0.0234
1	MOK023	MOK043	*TNFα*	0.0017
1	MOK023	MOK124	*TNFα*	0.026
1	MOK023	MOK128	*TNFα*	0.0432
1	MOK028	MOK043	*TNFα*	0.0112

Significant differences in IL-6 protein expression in primary bMEC infected with *S. aureus*

Time	Strain	Strain	Protein	Adj P
3	MOK006	MOK128	IL-6	0.0041
3	MOK008	MOK023	IL-6	0.0137
3	MOK008	MOK028	IL-6	0.007
3	MOK008	MOK032	IL-6	0.0007
3	MOK008	MOK098	IL-6	0.0001
3	MOK008	MOK128	IL-6	<.0001
3	MOK023	MOK124	IL-6	0.0149
3	MOK023	MOK128	IL-6	0.0373
3	MOK028	MOK076	IL-6	0.0353
3	MOK028	MOK083	IL-6	0.0379
3	MOK028	MOK124	IL-6	0.0077
3	MOK032	MOK043	IL-6	0.0064
3	MOK032	MOK076	IL-6	0.0041
3	MOK032	MOK077	IL-6	0.0078
3	MOK032	MOK083	IL-6	0.0044
3	MOK032	MOK124	IL-6	0.0008
3	MOK043	MOK098	IL-6	0.0012
3	MOK043	MOK128	IL-6	<.0001
3	MOK076	MOK098	IL-6	0.0008
3	MOK076	MOK128	IL-6	<.0001
3	MOK077	MOK098	IL-6	0.0015
3	MOK077	MOK128	IL-6	<.0001
3	MOK083	MOK098	IL-6	0.0008
3	MOK083	MOK128	IL-6	<.0001
3	MOK098	MOK124	IL-6	0.0001
3	MOK124	MOK128	IL-6	<.0001
6	MOK006	MOK098	IL-6	0.0045
6	MOK008	MOK098	IL-6	<.0001
6	MOK023	MOK098	IL-6	<.0001
6	MOK023	MOK128	IL-6	0.0056
6	MOK028	MOK098	IL-6	<.0001
6	MOK028	MOK128	IL-6	0.0119
6	MOK032	MOK098	IL-6	<.0001
6	MOK032	MOK128	IL-6	0.0119
6	MOK043	MOK098	IL-6	0.0003
6	MOK076	MOK098	IL-6	<.0001
6	MOK076	MOK128	IL-6	0.0119
6	MOK077	MOK098	IL-6	<.0001
6	MOK077	MOK128	IL-6	0.0119
6	MOK083	MOK098	IL-6	<.0001
6	MOK083	MOK128	IL-6	0.0119
6	MOK098	MOK124	IL-6	<.0001
6	MOK124	MOK128	IL-6	0.0056

Significant differences in IL-8 protein expression in primary bMEC infected with *S. aureus*

Time	Strain	Strain	Protein	Adj P
6	MOK023	MOK076	IL-8	0.0379
6	MOK076	MOK124	IL-8	0.0141
